# Correction: The magnitude of hypertension and associated factors among clients on highly active antiretroviral treatment in Southern Ethiopia, 2020: A hospital-based cross-sectional study

**DOI:** 10.1371/journal.pone.0274985

**Published:** 2022-09-15

**Authors:** The PLOS ONE Staff

The fourth author’s name is spelled incorrectly. The correct name is: Aklilu Habte.

The publisher apologizes for the error.
