# Supplementary material for: The magnitude of hypertension and associated factors among clients on highly active antiretroviral treatment in Southern Ethiopia, 2020: A hospital-based cross-sectional study
Source: PLoS One. 2021 Oct 15;16(10):e0258576. doi: 10.1371/journal.pone.0258576 (PMC8519467; doi:10.1371/journal.pone.0258576)
Supplement: S1 Questionnaire — (DOCX) [file pone.0258576.s002.docx]

## **DATA COLLECTION TOOL TO ASSESS THE MAGNITUDE OF HYPERTENSION AND ASSOCIATED FACTORS AMONG CLIENTS RECEIVING HIGHLY ACTIVE ANTIRETROVIRAL TREATMENT (HAART) IN SOUTHERN ETHIOPIA**

| **Sr.No** | **Questions** | **Response and coding** | **Skip** | **Remark** |
| --- | --- | --- | --- | --- |
| **100** | **Socio-demographic factors** |  |  |  |
| 101 | Age in completed years | ______yr |  |  |
| 102 | Sex | 1. Male 2. Female |  |  |
| 103 | What is your Ethnicity | 1. Hadiya 2. Kembata 3. Silte 4. Gurage 5. Amhara 6. Oromo 7. Other specify... |  |  |
| 104 | What is your religion? | 1. Protestant 2. Orthodox 3. Muslim 4. Catholic 5. Others (specify)………. |  |  |
| 105 | Residence | 1. Urban 2. Rural |  |  |
| 106 | What is your current marital status? | 1. Single 2. Married 3. Divorced 4. Widowed |  |  |
| 107 | What is your current occupation? | 1. Housewife 2. Farmer 3. Merchant 4. Government employee 5. Daily Laborer 6. Student 7. Driver 8. CSW 9. housemaid 10. others(specify)……. |  |  |
| 108 | What is your educational status or level? | 1. No education 2. Primary education(1-8) 3. Secondary (9-12) 4. Certificate 5. Diploma 6. Degree and above |  |  |
| 109 | What is your average monthly income | _______________(Birr) |  |  |
|  |  |  |  |  |
| **200** | **Clinical related Characteristics** | | | |
| 204 | Lipodystrophy | 1. Yes____ 0. No___ |  |  |
| 205 | Have you ever been measured your blood pressure? | 1. Yes____ 0. No___ |  |  |
| 206 | If yes to question 205, do you have hypertension? | 1. Yes____ 0. No___  3. I don’t know |  |  |
| 207 | Are you on follow up? | 1. Yes____ 0. No___ |  |  |
| 208 | If yes to question 207, what is the reason for your follow up? | _________________ |  |  |
| 209 | If no to question 207, what’s the reason? | _____________________ |  |  |
| 210 | Are you on drug treatment? | 1. .Yes____ 0. No___ |  |  |
| 211 | If yes to question 210, what is the reason? | ________________ |  |  |
| 212 | If no to question 210, what’s the reason? | _____________________ |  |  |
| 214 | How long have you been on HAART (in months)? | _________________ |  |  |
| 215 | Was there a regimen change? | 1. Yes 0. No |  |  |
| 216 | If yes to question no. 215 how many times? | __________________ |  |  |
| 219 | Do you take your HAART regimens in the preceding 7 days without interruption? (even without missing a single dose) | 1. Yes ____ 0. No_____ |  |  |
| 220 | If no to question no.219, what was the reason for not taking the regimen? | __________________ |  |  |
| 223 | Family history of Non-communicable diseases? | 1. Yes______ 0. No______   3.I don’t know |  |  |
| 224 | If yes to question no. 223, what was the chronic disease? | 1. Cardiovascular disorders 2. DM 3. Kidney diseases 4. Others specify________ |  |  |
| 225 | Have you ever been treated for Chronic diseases? | 1. Yes______ 0. N0____ |  |  |
| 226 | If yes to question no. 225, what was the reason for treatment? | 1. Cardiovascular disorders 2. DM 3. TB 4. Kidney diseases 5. Others specify________ |  |  |
| **300Behavioral, Diet, and Life style related factors** | | | | |
|  | Diet-related characteristics | |  |  |
| 301 | In a typical week, how often do you eat fruit? | _________servings /day.  _________times/a week |  |  |
| 302 | In a typical week, how often do you eat vegetables? | _________servings /day.  _________times/day |  |  |
| 303 | What type of oil/ butter/ is most often used for meal preparation in your household? | 1. Saturated 2. Unsaturated 3. Butter |  |  |
| 304 | On average, how many meals per week do you eat that was not prepared at a home? By meal, I mean breakfast, lunch, and dinner. | ________days/wk. |  |  |
| 305 | How many teaspoons of salt do you add to your food? | __________teaspoons |  |  |
| Behavior related characteristics | | | | |
| 306 | Do you currently smoke tobacco? | 1. Yes____ 0. No___ |  |  |
| 307 | If yes to question no. 306, how often? | 1. Daily 2. Less than daily 3. Not at all 4. Don’t know |  |  |
| 308 | Have you smoked tobacco in the past? | 1. Yes____ 0. No___  2. Don’t know |  |  |
| 309 | If yes to question no. 308, how often? | 1. Daily 2. Less than daily 3. Not at all 4. Don’t know |  |  |
| 310 | Have you ever chewed chat? | 1. Yes____ 0. No___ |  |  |
| 311 | If yes to question no.310, how often have you chewed chat in the last 30 days? | _______times/day or _______times/week |  |  |
| 312 | How often do you have a drink containing alcohol? | 1. Never 2. Monthly or less than 2 to 4 times a month 3. 2 to 4 times a month 4. 2 to 3 times a week 5. 4 or more times a week |  |  |
| 313 | What is the type of alcohol you frequently drink? | 1. Tej 2. Tella 3. Aereke /katikala/ 4. Beer 5. Other specify……… |  |  |
| 314 | How many drinks containing that alcohol do you have on a typical day when you are drinking? | 1. 1or 2 2. 3 or 4 3. 5 or 6 4. 7, 8 or 9 5. 10 or more |  |  |
| 315 | How often do you have six or more drinks on one occasion? | 1. Never 2. Less than monthly 3. Monthly 4. Weekly 5. Daily or almost daily |  |  |
| 316 | How often during the last year have you found that you were not able to stop drinking once you had started? | 1. Never 2. Less than monthly 3. Monthly 4. Weekly 5. Daily or almost daily |  |  |
| 317 | How often during the last year have you failed to do what was normally expected from you because of drinking? | 1. Never 2. Less than monthly 3. Monthly 4. Weekly 5. Daily or almost daily |  |  |
| 318 | How often during the last year have you needed a first drink in the morning to get yourself going after a heavy drinking session? | 1. Never 2. Less than monthly 3. Monthly 4. Weekly 5. Daily or almost daily |  |  |
| 319 | How often during the last year have you had a feeling of guilt or remorse after drinking? | 1. Never 2. Less than monthly 3. Monthly 4. Weekly 5. Daily or almost daily |  |  |
| 320 | How often during the last year have you been unable to remember what happened the night before because you had been drinking? | 1. Never 2. Less than monthly 3. Monthly 4. Weekly 5. Daily or almost daily |  |  |
| 321 | Have you or someone else been injured as a result of your drinking? | 1. Yes but during the last year 2. Yes but not during the last year 3. No |  |  |
| 322 | Does your work involve a vigorous-intensity activity that causes large increases in breathing or heart rate like [carrying or lifting heavy loads, digging, or construction work] for at least 10 minutes continuously? | 1. Yes_____ 0. No______ |  |  |
| 323 | If yes to question no.323, In a typical week, on how many days do you do vigorous-intensity activities as part of your work? | _________________ days |  |  |
| 324 | How much time do you spend doing vigorous-intensity activities at work on a typical day? | _____________hrs, or ______________minutes |  |  |
| 325 | If your answer is no to question no.322, does your work involve moderate-intensity activity, that causes small increases in breathing or heart rate such as brisk walking [or carrying light loads] for at least 10 minutes continuously? | 1. Yes_____ 0. No______ |  |  |
| 326 | If yes to question no.325 In a typical week, on how many days do you do moderate-intensity activities as part of your work? | _____________________ days |  |  |
| 327 | If yes to question no, how much time do you spend doing moderate-intensity activities at work on a typical day? | _____________minutes or _____________hrs |  |  |
| 328 | Do you walk or use a bicycle (pedal cycle) for at least 10 minutes continuously to get to and from places? | 1. Yes_____ 0. No______ |  |  |
| 329 | In a typical week, on how many days do you walk or bicycle for at least 10 minutes continuously to get to and from places? | __________________ days |  |  |
| 330 | How much time do you spend walking or bicycling for travel on a typical day? | _____________ minutes or _________ hrs |  |  |
| 331 | Do you do any vigorous-intensity sports, fitness, or recreational (leisure) activities that cause large increases in breathing or heart rate like [running or football] for at least 10 minutes continuously? | 1. Yes_____ 0. No______ |  |  |
| 332 | In a typical week, on how many days do you do vigorous-intensity sports, fitness, or recreational (leisure) activities? | ________________ days |  |  |
| 333 | How much time do you spend doing vigorous-intensity sports, fitness, or recreational activities on a typical day? | _______________ minutes or ____________ hrs |  |  |
| 334 | Do you do any moderate-intensity sports, fitness, or recreational (leisure) activities that cause a small increase in breathing or heart rate such as brisk walking, [cycling, swimming, volleyball] for at least 10 minutes continuously? | 1. Yes_______ 0. N0________ |  |  |
| 335 | In a typical week, on how many days do you do moderate intensity sports, fitness or recreational (leisure) activities | ____________________ days |  |  |
| 336 | How much time do you spend doing moderate-intensity sports, fitness, or recreational (leisure) activities on a typical day? | ________________ minutes or ________________ hrs |  |  |
| 337 | How much time do you usually spend sitting or reclining on a typical day? | ________________ minutes or ________________ hrs |  |  |
| Stress-Related Questions | | | | |
| 338 | In the last month, how often have you been upset because of something that happened unexpectedly? | 0 = Never  1 = Almost Never  2 = Sometimes  3 = Fairly Often  4 = Very Often |  |  |
| 339 | In the last month, how often have you felt that you were unable to control the important things in your life? | 0 = Never  1 = Almost Never  2 = Sometimes  3 = Fairly Often  4 = Very Often |  |  |
| 340 | In the last month, how often have you felt nervous and “stressed”? | 0 = Never  1 = Almost Never  2 = Sometimes  3 = Fairly Often  4 = Very Often |  |  |
| 341 | In the last month, how often have you felt confident about your ability to handle your personal problems? | 0 = Never  1 = Almost Never  2 = Sometimes  3 = Fairly Often  4 = Very Often |  |  |
| 342 | In the last month, how often have you felt that things were going your way? | 0 = Never  1 = Almost Never  2 = Sometimes  3 = Fairly Often  4 = Very Often |  |  |
| 343 | In the last month, how often have you found that you could not cope with all the things that you had to do? | 0 = Never  1 = Almost Never  2 = Sometimes  3 = Fairly Often  4 = Very Often |  |  |
| 344 | In the last month, how often have you been able to control irritations in your life? | 0 = Never  1 = Almost Never  2 = Sometimes  3 = Fairly Often  4 = Very Often |  |  |
| 345 | In the last month, how often have you felt that you were on top of things? | 0 = Never  1 = Almost Never  2 = Sometimes  3 = Fairly Often  4 = Very Often |  |  |
| 346 | In the last month, how often have you been angered because of things that were outside of your control? | 0 = Never  1 = Almost Never  2 = Sometimes  3 = Fairly Often  4 = Very Often |  |  |
| 347 | In the last month, how often have you felt difficulties were piling up so high that you could not overcome them? | 0 = Never  1 = Almost Never  2 = Sometimes  3 = Fairly Often  4 = Very Often |  |  |
| Knowledge about the prevention of hypertension | | | | |
| 348 | What is the normal range of blood pressure? | 1. 90/60 mmHg 2. 120/80 mmHg 3. 140/90 mmHg 4. I don’t know |  |  |
| 349 | What is meant by hypertension? | 1. Blood pressure measure of >= 140/90 mmHg 2. Blood pressure measure of 120/80mmHg 3. Blood pressure measure of 90/60mmHg 4. I don’t know |  |  |
| 350 | What is the risk factor of hypertension among the questions given below (more than one answer is possible)? | 1. Stress 2. Age 3. Diet 4. Smoking 5. Physical inactivity 6. Cigarette smoking 7. Hereditary 8. Don’t know |  |  |
| 351 | Did the disease hypertension have signs and symptoms most of the time? | 1. Yes _______ 2. No ________ |  |  |
| 352 | What damage to major organs may be caused by hypertension? | 1. Liver dysfunction 2. Gastrointestinal Dysfunction 3. Respiratory Dysfunction 4. Renal Dysfunction 5. Don’t know |  |  |
| 353 | What is the appropriate diagnostic study of hypertension? | 1. Blood test 2. Urine test 3. Chest x-ray 4. Blood pressure measurement 5. Don’t know |  |  |
|  |  |  |  |  |
| Attitude about prevention of Hypertension | | | | |
| 354 | Hypertension is preventable. | 1. Strongly agree 2. Agree 3. Uncertain   Disagree   1. Strongly disagree |  |  |
| 355 | Stopping cigarette smoking and alcohol drinking helps to prevent hypertension. | 1. Strongly agree 2. Agree 3. Uncertain 4. Disagree 5. Strongly disagree |  |  |
| 356 | It is good to avoid extra added salts in your diet. | 1. Strongly agree 2. Agree 3. Uncertain 4. Disagree 5. Strongly disagree |  |  |
| 357 | It is good to have whole fruits rather than to have deserts and sweets. | 1. Strongly agree 2. Agree 3. Uncertain 4. Disagree 5. Strongly disagree |  |  |
| 358 | BP should be checked periodically. | 1. Strongly agree 2. Agree 3. Uncertain 4. Disagree 5. Strongly disagree |  |  |
| 359 | Regular exercise helps to prevent hypertension. | 1. Strongly agree 2. Agree 3. Uncertain 4. Disagree 5. Strongly disagree |  |  |
| 360 | The hypertensive patient can take fat-rich food | 1. Strongly agree 2. Agree 3. Uncertain 4. Disagree 5. Strongly disagree |  |  |
| 361 | Hypertensive patients should keep away from the stress-inducing situation. | 1. Strongly agree 2. Agree 3. Uncertain 4. Disagree 5. Strongly disagree |  |  |
| 400**Measurements** | | | | |
| 401 | Weight (Kg) | ________________ |  |  |
| 402 | Height (M) | ________________ |  |  |
| 403 | Waist Circumference (cm) | _______________ |  |  |
| 404 | Hip Circumference (cm) | ________________ |  |  |
| 405 Blood Pressuremeasure | | | | |
|  | Reading 1 | Systolic …………( mmHg)  Diastolic……….. (mmHg) |  |  |
|  | Reading 2 | Systolic …………( mmHg)  Diastolic……….. (mmHg) |  |  |

**English Version Data Extraction tool**

NB. The following questions are to be filled from the client chart.

| **Sr.No** | **Questions** | **Response and coding** | **Skip** | **Remark** |
| --- | --- | --- | --- | --- |
| Clinical Related Characteristics | | | | |
| 201 | Recent WHO clinical stage? | 1. Stage I 2. Stage II 3. Stage III 4. Stage IV |  |  |
| 202 | CD4 count baseline? | __________cells/mm3 |  |  |
| 203 | Is CD4 count recent? | __________cells/mm3 |  |  |
| 213 | Currently used regimen? | 1. AZT/3TC/NVP 2. AZT/3TC/EFV 3. TDF/3TC/NVP 4. TDF/3TC/EFV 5. Others ________________ |  |  |
| 217 | Duration of each regimen (in months)? | __________________ |  |  |
| 218 | Mostly used HAART regimen? | 1. d4T/3TC/NVP 2. d4T/3TC/EFV 3. AZT/3TC/NVP 4. AZT/3TC/EFV 5. TDF/3TC/NVP 6. TDF/3TC/EFV 7. Others ________________ |  |  |
| 221 | Opportunistic Infection? | 1. Yes_____ 0. No_____ |  |  |
| 222 | If yes to question no.221, what was the diagnosed opportunistic infection? | 1. Skin Disorders 2. TB 3. HBV 4. HCV 5. Others specify______ |  |  |
| 228 | recent viral load | ____________copies/ml |  |  |
| 229 | HDL recent | ________________mg/dl |  |  |
| 230 | LDL recent | ________________mg/dl |  |  |
| Baseline Anthropometric Characteristics | | | | |
| 401 | Weight baseline | ___________Kg |  |  |

Name of data collector_________________________________

Signature _______________________

Date __________________________

Annex III የአማርኛመረጃእናስምምንትመግለጫ

ቀን.................................ሰዓት..........................የቃለመጠይቅመለያቁጥር.............................

እንደምንአደሩ/ዋሉ?

ስሜ.......................................................ይባላልየስራባልደረባዬደግሞቃለእግዚአብሄር ሉቃስይባላሉ ;; በሃዋሳ ዩኒቨርሲቲየህብረተሰበጤናሳይንስትምህርትቤትየ ሁለተኛ ዲግሪ ተመራቂ ተማሪናቸዉ፡፡ ዛሬበዚህ የተገኘነው በ ንግስት እሌኒ መሃመድ መታሰቢያ ሆስፒታል ጸረ ኤች አይ ቪ መድሃኒት ተጠቃሚዎች መሃል የ ደምግፊት ስርጭት መጠን እና ተያያዥ ጉዳዮችን ለመገምገምየሚያስችልመረጃለመሰበሰብነዉ፡፡የ ምርምሩ ውጤት የ ጸረ ኤች አይ ቪ ህክምናን ክፍተቶች ለ መሙላት እና የተሻለ ህክምና እንዲኖር እንዲያግዝ የታሰበ ነው፡፡

የሚሰበሰበዉመረጃሙሉበሙሉበሚስጥርየሚያዝመሆኑንእናረጋግጥልዎታለን;; የእርስዎስም፤መለያአድራሻአይመዘገብም;; መረጃመስጠትካልፈለጉመብትዎነዉ;; መመለስያልፈለጉትንጥያቄመዝለል/ማለፍ/ ይችላሉ;; ይሁንእንጂየእርስዎትብብርእናትክክለኛምላሽጥናቱናምርምሩእንዲሳካትልቅአስተዋጽኦይኖረዋል;; ስለዚህለሚቀርብልዎትጥያቄትክክለኛመልስለመስጠትፍቃደኛሆነዉበትዕግስትእንዲመልሱልንእንጠይቅዎታለን፡፡

ቃለመጠይቁበግምት 30 ደቂቃይፈጃል፡፡

በጥናቱውስጥለመሳተፍፍቃደኛነዎት?

አዎአይደለሁም

የመረጃሰብሳቢዉስም --------------------------------------------- ፊርማ ----------------------

የተቆጣጣሪዉስም --------------------------------------------------- ፊርማ ----------------------

ለ በለጠ መረጃ፡ የተመራማሪው ስም፡ ቃለእግዚአብሄር ሉቃስ ስልክ 0916286878

ኢሜይል: kaleegziabherad12@gmail.com

# Annex Ivየአማርኛመጠይቅ

የመጠይቅመለያ______

| **ተ.ቁ** | ጥያቄዎች | መልስ | ዝለል | ምርመራ |
| --- | --- | --- | --- | --- |
| **100** | **አጠቃላይመረጃ** |  |  |  |
| 101 | ሙሉ ዕድሜዎ ስንት ነው? | ______ዓመት |  |  |
| 102 | ጾታ | 1. ወንድ 2. ሴት |  |  |
| 103 | ብሔረሰብ | 1. ሀድያ 2. ከንባታ 3. ስልጤ 4. ጉራጌ 5. ሌላ______ |  |  |
| 104 | ሀይማኖት | 1. ፕሮቴስታንት 2. ኦርቶዶክስ 3. ሙስሊም 4. ካቶሊክ 5. ሌላ______ |  |  |
| 105 | መኖሪያዎ የትነው? | 1. ከተማ 2. ገጠር |  |  |
| 106 | በአሁኑ ጊዜ የጋብቻ ሁኔታዎ ምንድነው? | 1. ያላገባ 2. ያገባ 3. የተፋታ 4. ባል/ሚስትየሞተበት |  |  |
| 107 | በአሁኑ ጊዜ በምን ስራ ነው ሚተዳደሩት? | 1. የ ቤት እመቤት 2. አርሶአደር 3. ነጋዴ 4. የ መንግስትሠራተኛ 5. የ ቀን ሰራተኛ 6. የ ግልተዳዳሪ 7. ተማሪ 8. የቤት ሰራተኛ 9. ሌላ (ጥቀስ)……. |  |  |
| 108 | የትምህርትደረጃዎምንድንነው? | 1. አልተማርኩም 2. የመጀመሪያደረጃት/ቤት (1-8) 3. ሁለተኛደረጃት/ቤት (9-12) 4. ከሁለተኛደረጃት/ቤትበላይ (9-12) 5. ሰርተፊኬት 6. ዲፕሎማ 7. ዲግሪ እና ከዚያ በላይ |  |  |
| 109 | ወርሃዊ ገብይዎ በ አማካይ ምንያህልነው? | _____ ብር |  |  |
| **ከህክምናጋርየተያያዙ መጠይቆች** | | | |  |
| 204 | ከጸረኤችአይቪ/ /መድሀኒት/ጋርተያያዥነትያለውጤነኛያልሆነ ስብክምችት | 1.አዎን____ 0.አይደለም___ |  |  |
| 205 | የ ደም ግፊት መጠንዎን ተለክተው ያውቃሉ? | 1.አዎን____ 0.አይደለም___ |  |  |
| 206 | ለጥያቄ ቁጥር 205 መልስዎ አዎን ከሆነ፡ የ ደም ግፊት አለብዎት? | 1.አዎን____ 0.አይደለም___  3. አላውቅም |  |  |
| 207 | የህክምና ክትትል ያደርጋሉ? | 1.አዎን____ 0.አይደለም___ |  |  |
| 208 | ለጥያቄ ቁጥር 207 መልስዎ አዎን ከሆነ ፡ ለምን ህመም ነበር ክትትል የሚያደርጉት? | ­­­ ________________ |  |  |
| 209 | ለጥያቄ ቁጥር 207 መልስዎ አይደለም ከሆነ፤ ምክንያትዎ ምን ነበር? |  |  |  |
| 210 | መድሃኒትዎን ይወስዳሉ? | 1. አዎን____ 0.አይደለም___ |  |  |
| 211 | ለጥያቄ ቁጥር 210 መልስዎ አዎን ከሆነ፤ መድሃኒት የሚወስዱት ለምን ህመም ነበር? |  |  |  |
| 212 | ለጥያቄ ቁጥር 210 መልስዎ አይደለም ከሆነ፤ በምን ምክኒያት ነበር መድሃኒትዎን የማይወስዱት? |  |  |  |
| 214 | የ ጸረ ኤች አይ ቪ መድሃኒት መውሰድ ከጀመሩ ም ያህል ጊዜ ሆንዎት (በወራት)? |  |  |  |
| 215 | የ ጸረ ኤች አይ ቪ መድሃኒትዎን ቀይረው ያውቃሉ? | 1. አዎን____ 0.አይደለም___ |  |  |
| 216 | ለጥያቄ ቁጥር 215 መልስዎ አዎን ከሆነ፤ መድሃኒት የቀየሩበት ምክንይት ምን ነበር? |  |  |  |
| 219 | ባለፉት ሰባት ቀናት የ ጸረ ኤች አይ ቪ መድሃኒትዎን ያለማቋረጥ ውሰደዋልን? (ይህ ማለት አንድም ጊዜ ሳያቋርጡ ?) | 1. አዎን____ 0.አይደለም___ |  |  |
| 220 | ለጥያቄ ቁጥር 219 መልስዎ አይደለም ከሆነ፤ የ ጸረ ኤች አይ ቪ መድሃኒትዎን ያለማቋረጥ ያልወሰዱበት ምክንይት ምን ነበር? |  |  |  |
| 223 | ከቤተሰብዎ መካከል ተላላፊ ያለሆኑ በሽታዎች ህመተኛ አለ? | 1. አዎን____ 0.አይደለም___   3.አላውቅም |  |  |
| 224 | ለጥያቄ ቁጥር 223 መልስዎ አዎን ከሆነ፤ ተላላፊ ያለሆነው የ ህመም አይነት ምን ነበር? | 1. የ ልብ ህመም 2. የ ስኳር ህመም 3. የ ኩላሊት 4. ሌላ---------- |  |  |
| 225 | ለ ረዥም ጊዜ ለህመም ለሚዳርጉ የ ህመም አይነቶች ህክምና አድርገው ያውቃሉ? | 1. አዎን____ 0.አይደለም___   3.አላውቅም |  |  |
| 226 | ለጥያቄ ቁጥር 226 መልስዎ አዎን ከሆነ፤ ህክምና እንዲወስዱ የዳረግዎ ለረዥም ጊዜ ለህመም የሚዳርገው የ ህመም አይነት ምን ነበር? | 1. የ ልብ ህመም 2. የ ስኳር ህመም 3. የ ኩላሊት ህመም 4. ነቀርሳ 5. ሌላ---------- |  |  |
|  |  |  |  |  |
| 300 **ከባህሪይ አመጋገብ ዘዬ እና አኗኗር ሁኔታዎች ጋርየተያያዙ ጥያቄዎች** | | | | |
| ከ አመጋገብ ሁኔታ ጋር ተያያዥነት ያላቸው መጠይቖች | | | |  |
| 301 | በቀን ምንያህል ጊዜ ፍራፍሬ ይመገባሉ? | __________ ጊዜ በቀን  __________በሳምንት |  |  |
| 302 | በቀን ምንያህል ጊዜ አትክልቶችንይመገባሉ? | __________ ጊዜ በቀን  __________በሳምንት |  |  |
| 303 | አብዛኛውን ጊዜ ምግብዎን ለማብሰል የሚጠቀሙበት ዘይት ምንድነው? | 1. የሚረጋ ዘይት 2. የማይረጋ ዘይት 3. ቅቤ |  |  |
| 304 | በሳምንት በ አማካይ ምንያህል ጊዜ ከቤት ውጪ ይመገባሉ? | -----------------ቀናት በሳምንት |  |  |
| 305 | በቀን ምን ያህል ጨው በምግብዎ ውስጥ ይጠቀማሉ? | ___ የሻይ ማንኪያ በቀን |  |  |
| ከ ባህሪ ጋር ተያያዝነት ያላቸው መጠይቆች | | | |  |
| 306 | በ አሁኑ ጊዜ ሲጋራ ያጨሳሉ? | 1.አዎን 0.አይደለም |  |  |
| 307 | ለጥያቄ ቁጥር 306 መልስዎ አዎን ከሆነ፤ ሲጋራ ማጬስን ምን ያህል ያዘወትራሉ? | 1. በ የዕለቱ 2. አንዳንድ ጊዜ 3. በጭራሽ 4. አላውቅም |  |  |
| 308 | ከዚህ ቀደም ሲጋራ ያጬሱ ነበር? | 1.አዎን ____ 0.አይደለም___  3. አላውቅም |  |  |
| 309 | ለጥያቄ ቁጥር 308መልስዎ አዎን ከሆነ፤ ሲጋራ ማጬስን ምን ያህል ያዘወትራሉ? | 1. በ የዕለቱ 2. አንዳንድ ጊዜ 3. በጭራሽ 4. አላውቅም |  |  |
| 310 | ጫት ቅመው ያውቃሉ? | 1.አዎን ____ 0.አይደለም___  3. አላውቅም |  |  |
| 311 | ለጥያቄ ቁጥር 310መልስዎ አዎን ከሆነ፤ ባለፉት 30 ቀናት ጫት ቅመው ያውቃሉ? | ------------ጊዜ በቀን ወይም -------ጊዜ በሳምንት |  |  |
| 312 | አልኮል ያለበት መጠጥ ምንያህል አዘውትረው ያውቃሉ? | 1. በጭራሽ 2. በየወሩ ወይም በ ወር ከ 2 እስከ 4 ጊዜ በታች 3. በ ወር ከ 2 እስከ 4 ጊዜ 4. በ ሳምንት ከ 2 እስከ 3 ጊዜ 5. በ ሳምንት 4 ወይም ክ አራት ጊዜ በላይ |  |  |
| 313 | የትኛውን አይነት አልኮል መጠጥ ነው አዘውትረው ሚጠጡት? | 1. ጠጅ 2. ጠላ 3. አረቄ /ካቲካላ/ 4. ቢራ 5. ሌላ ይጥቀሱ…….. |  |  |
| 314 | እረስዎ የ ሚያዘውትሩትን አልኮል መጠጥ በ ቀን ምን ያህል ይጠጣሉ? | 1. ከ 1 እስከ 2 2. ከ 3 እስከ 4 3. ከ 5 እስከ 6 4. ከ 7 እስከ 9 5. ከ 10 በላይ |  |  |
| 315 | በ አንድ ጊዜ ከ 6 በላይ አልኮል የመጠጣትን ም ያህል ያዘወትራሉ? | 1. በ ጭራሽ 2. ከ ወር ባነሰ ሁኔታ 3. በ የወሩ 4. በ የሳምንቱ 5. በ የቀኑ |  |  |
| 316 | ባለፉት 12 ወራት መጠጥ ለማቆም ወስነው ምን ያህል ጊዜ ተቸግረው ያውቃሉ? | 1. በ ጭራሽ 2. ከ ወር ባነሰ ሁኔታ 3. በ የወሩ 4. በ የሳምንቱ 5. በ የቀኑ |  |  |
| 317 | ባለፉት 12 ወራት በመጠጥ ምክኒያት ማድረግ ያለቦትን ነገር ምን ያህል ሳያደርጉ ቀርተው ያውቃሉ? | 1. በ ጭራሽ 2. ከ ወር ባነሰ ሁኔታ 3. በ የወሩ 4. በ የሳምንቱ 5. በ የቀኑ |  |  |
| 318 | ባለፉት 12 ወራት ምን ያህል ጊዜ በ ጥዋት መጠጥ መጠጣት ጀምረው ያውቃሉ? | 1. በ ጭራሽ 2. ከ ወር ባነሰ ሁኔታ 3. በ የወሩ 4. በ የሳምንቱ 5. በ የቀኑ |  |  |
| 319 | ባለፉት 12 ወራት መጠጥ በመጠጣትዎ ምክኒያት ምንያያል ጊዜ የ ጥፋተኘነት ስሜት / ተጸጽተው ያውቃሉ? | 1. በ ጭራሽ 2. ከ ባነሰ ሁኔታ 3. በ የወሩ 4. በ የሳምንቱ 5. በ የቀኑ |  |  |
| 320 | መጠጥ በመጠጣትዎ ምክንያት እርስዎ ላይ ወይም ሌላ ሰው ላይ ጉዳት ደርሶ ያውቃል? | 1. ያለፈው አመት፤ አዎን 2. በ ዚህ አመት ፤ አዎን 3. በ ጭራሽ |  |  |
| 321 | ስራዎ ከባድ ሥራ እንደ መሸከም ከባድ ነገር ማንሳት ማለትም የ ልብ ምትዎን እና አተነፋፈስ ሁኔታዎችን በከፍተኛኛ ደረጃ የሚጨምሩ ወይንም እንደ ግንባታ ሥራ ቢያንስለ 10 ደቂቃ ያክል በቀጣይነት ያካተተ ነውን? | 1. አዎን____ 0.አይደለም___ |  |  |
| 322 | ለ ጥያቄ ቁጥር 321 መልስዎ አዎን ከሆነ እንዲህ አይነት ከባድ ስራዎችን በሳምንት ምን ያክል ጊዜ ትሰራለህ? | _________ቀናት |  |  |
| 323 | ከባድ ስራዎችን በቀን ምን ያክል ጊዜ ይሰራሉ? | _________ ሰአት _________ደቂቃ |  |  |
| 324 | ለጥያቄ ቁጥር 321መልስዎ አይደለም ከሆነ፡ መጠነኛ የሆኑ ስረዎች ማለትም የ ልብ ምትዎን እና አተነፋፈስ ሁኔታዎችን በመጠነኛ ደረጃ የሚጨምሩ ስራዎችን ለ 10 ደቂቃ በ ቀጣይነት ይሰራሉ? | 1. አዎን____ 0.አይደለም___ |  |  |
| 325 | ለ ጥያቄ ቁጥር 324 መልስዎ አዎን ከሆነ እንዲህ አይነት መጠነኛ ስራዎችን በሳምንት ምን ያክል ጊዜ ይሰራሉ? | _________ቀናት |  |  |
| 326 | መጠነኛ ስራዎችን በቀን ምን ያህል ጊዜ ይሰራሉ? | _________ ሰአት _________ደቂቃ |  |  |
| 327 | ከ ቦታ በታ ለምንቀሳቀስ ብስክሌት ተሸከርካሪ ቢያንስ ለ 10 ደቂቃ በቀጣይነት ይጠቀማሉ? | 1. አዎን____ 0.አይደለም___ |  |  |
| 328 | በሳምንት ከ ቦታ ቦታ ለመንቀሳቀስ ምን ያህል ቀናትን በ እግር ለ 10 ደቂቃ በ ቀጣይነት እርምጃ ያደርጋሉ? | _________ቀናት |  |  |
| 329 | በቀን ከ ቦታ ቦታ ለመንቀሳቀስ ምን ያህል ጊዜ በ እግር ለ 10 ደቂቃ በ ቀጣይነት እርምጃ ያደርጋሉ? | _________ ሰአት _________ደቂቃ |  |  |
| 330 | በ ትርፍ ጊዜዎ ወይም ስፖርት በሚሰሩበት ወቅት የ ሚሰሩት የ ሰውነት እንቅስቃሴ ከባድ ነገር እንደ ማንሳት ማለትም የ ልብ ምትዎን እና አተነፋፈስ ሁኔታዎችን በከፍተኛኛ ደረጃ የሚጨምሩ ስራዎችን ይሰራሉን? | 1. አዎን____ 0.አይደለም___ |  |  |
| 331 | በ ትርፍ ጊዜዎ ወይም ስፖርት በሚሰሩበት ወቅት ምን ያህል ቀናትን በሳምንት ከባድ እንቅስቃሴዎችን ያደርጋሉ? | _________ቀናት |  |  |
| 332 | በ ትርፍ ጊዜዎ ወይም ስፖርት በሚሰሩበት ወቅት ምን ያህል ጊዜ በቀን ከባድ እንቅስቃሴዎችን ያደርጋሉ? | _________ ሰአት _________ደቂቃ |  |  |
| 333 | በ ትርፍ ጊዜዎ ወይም ስፖርት በሚሰሩበት ወቅት የ ሚሰሩት የ ሰውነት እንቅስቃሴ የ ልብ ምትዎን እና አተነፋፈስ ሁኔታዎችን መጠነኛ ሁኔታ የሚጨምሩ ስራዎችን ይሰራሉን? | 1. አዎን____ 0.አይደለም___ |  |  |
| 334 | በ ትርፍ ጊዜዎ ወይም ስፖርት በሚሰሩበት ወቅት ምን ያህል ቀናትን በሳምንት መጠነኛ እንቅስቃሴዎችን ያደርጋሉ? | _________ቀናት |  |  |
| 335 | በ ትርፍ ጊዜዎ ወይም ስፖርት በሚሰሩበት ወቅት ምን ያህል ጊዜ በቀን መጠነኛ እንቅስቃሴዎችን ያደርጋሉ? | _________ ሰአት _________ደቂቃ |  |  |
| 336 | በ ቀን ምን ያህል ጊዜ ቁጭ ብለው ያሳልፋሉ? | _________ ሰአት _________ደቂቃ |  |  |
| ጭንቀትን የተመለከቱ መጠይቆች | | | | |
| 337 | ባለፈው ወር፤ ያለታሰበ ክስተት ገጥሞት ምን ያህል ጊዜ ተበሳጭተው ውቃሉ? | 1. በፍጹም 2. በጣም አልፎ አልፎ 3. አንድ አንድ ጊዜ 4. ብዙ ጊዜ 5. በጣም ብዙ ጊዜ |  |  |
| 338 | ባለፈው ወር፤ በህይወትዎ ጠቃሚ ነገሮችን መቆጣጠር ሳይችሉ ምን ያህል ጊዜ ቀርተው የውቃሉ? | 1. በፍጹም 2. በጣም አልፎ አልፎ 3. አንድ አንድ ጊዜ 4. ብዙ ጊዜ 5. በጣም ብዙ ጊዜ |  |  |
| 339 | ባለፈው ወር፤ ምን የህል ጊዜ ተበሳጭተው እና ተጨንቀው ያውቃሉ? | 1. በፍጹም 2. በጣም አልፎ አልፎ 3. አንድ አንድ ጊዜ 4. ብዙ ጊዜ 5. በጣም ብዙ ጊዜ |  |  |
| 340 | ባለፈው ወር፤ ችግርዎን በራስዎ በመተማን ምን ያህል ጊዜ ሊፈቱ ችለዋል? | 1. በፍጹም 2. በጣም አልፎ አልፎ 3. አንድ አንድ ጊዜ 4. ብዙ ጊዜ 5. በጣም ብዙ ጊዜ |  |  |
| 341 | ባለፈው ወር፤ ነገሮች ምን ምን ያህል ጊዜ በራስዎ መነገድ አስኬዷቸው? | 1. በፍጹም 2. በጣም አልፎ አልፎ 3. አንድ አንድ ጊዜ 4. ብዙ ጊዜ 5. በጣም ብዙ ጊዜ |  |  |
| 342 | ባለፈው ወር፤ ባጋጠምዎት ሁኔታ ምንያህል ጊዜ ተጨንቀው ያውቃሉ? | 1. በፍጹም 2. በጣም አልፎ አልፎ 3. አንድ አንድ ጊዜ 4. ብዙ ጊዜ 5. በጣም ብዙ ጊዜ |  |  |
| 343 | ባለፈው ወር፤ የሚያበሳጩ ሁኔታዎችን ምንያህል ጊዜ መፍታት ችለው ነበር? | 1. በፍጹም 2. በጣም አልፎ አልፎ 3. አንድ አንድ ጊዜ 4. ብዙ ጊዜ 5. በጣም ብዙ ጊዜ |  |  |
| 344 | ባለፈው ወር፤ የ ገጠምዎትን ሁኔታዎች ሁሉ በ ምን ያህል ጊዜአግባቡ ሊፈቱ ችለዋል? | 1. በፍጹም 2. በጣም አልፎ አልፎ 3. አንድ አንድ ጊዜ 4. ብዙ ጊዜ 5. በጣም ብዙ ጊዜ |  |  |
| 345 | ባለፈው ወር፤ የሚያበሳጩ ሁኔታዎች ምን ያህል ጊዜ አጋጠሞት? | 1. በፍጹም 2. በጣም አልፎ አልፎ 3. አንድ አንድ ጊዜ 4. ብዙ ጊዜ 5. በጣም ብዙ ጊዜ |  |  |
| 346 | ባለፈው ወር፤ ከ ቁጥጥርዎ ውጪ በሆኑ ነገሮች ምን ያህል ጊዜ ተበሳጩ? | 1. በፍጹም 2. በጣም አልፎ አልፎ 3. አንድ አንድ ጊዜ 4. ብዙ ጊዜ 5. በጣም ብዙ ጊዜ |  |  |
| የ ደም ግፊት ህመም መከላከል ዕውቀትን የተመለከቱ ጥያቄዎች | | | | |
| 347 | የ አንድ ጤናማ ሰዉ የደም ግፊት መጠን ስንት በስንት ነዉ? | 1. 90/60ሚሜሜ 2. 120/80ሚሜሜ 3. 140/90 ሚሜሜ 4. አላዉቀዉም |  |  |
| 348 | የደም ግፊት በሽታ ማለት ምን ማለት ነዉ? | 1. የደሙ ግፊት 140/90 ሚሜሜ በላይ ሲሆን 2. የደሙ ግፊት 120/80ሚሜሜ ሲሆን 3. የደሙ ግፊት 90/50 ሚሜሜ ሲሆን 4. አላዉቀዉም |  |  |
| 349 | ለደም ግፊት በሽታ ተጋላጭነት መንስኤ የሆነዉ የቱ ነዉ? (ከ አንድ በላይ መልስ መመለስ ይችላሉ) | 1. ጭንቀት 2. እድሜ 3. የዘር ሀረግ 4. የ አካል ብቃት እንቅስቃሴ አለማድረግ 5. ሲጋራ ማጨ 6. አልኮል መጠጣት 7. ጤነኛ ያልሆን የኣመጋገብ ዘይቤ 8. አላዉቀዉም |  |  |
| 350 | የደም ግፊት በሽታ የህመም ምልክት ያሳያል ብለው ያስባሉ? | 1. አዎን 2. አይደለም |  |  |
| 351 | በደም ግፊት የሚጠቁ የሰዉነት ክፍሎች የትኞቹ ናቸዉ? | 1. የጉበት ተግባር መታወክ 2. የአንጀት ተግባር መታወክ 3. የመተንፈሻ አካላት መታወክ 4. የኩላሊት ተግባር መታወክ |  |  |
| 352 | የደምን ግፊት ለማወቅ የትኛው አይነት ምርመራ መደረግ አለበት? | 1. የደም ምርመራ 2. የሽንት ምርመራ 3. የደረት ራጅ ምርመራ 4. የደም ግፊትን መለካት 5. አላዉቀዉም |  |  |
| የ ደም ግፊት ህመም መከላከል አመለካከትን የተመለከቱ ጥያቄዎች | | | |  |
| 353 | የደም ግፊት በሽታን መከላከል ይቻላል፡፡ | 1. በጣም እስማማለሁ 2. እስማማለሁ 3. እርግጠኛ አይደለሁም 4. አልስማማም 5. በጣም አልስማማም |  |  |
| 354 | ሲጋራና አልኮልን ባለመጠቀም የደም ግፊት በሽታን መከላከል ይቻላል፡፡ | 1. በጣም እስማማለሁ 2. እስማማለሁ 3. እርግጠኛ አይደለሁም 4. አልስማማም 5. በጣም አልስማማም |  |  |
| 355 | በምግብ ላይ ተጨማሪ ጨዉ ያለመጠቀም ጥሩ ነዉ፡፡ | 1. በጣም እስማማለሁ 2. እስማማለሁ 3. እርግጠኛ አይደለሁም 4. አልስማማም 5. በጣም አልስማማም |  |  |
| 356 | ጣፋጭ ምግቦችን በመተዉ ፍራፍሬ ብቻ መጠቀም የደም ግፊት በሽታን ለመከላከል ጥሩ ነዉ፡፡ | 1. በጣም እስማማለሁ 2. እስማማለሁ 3. እርግጠኛ አይደለሁም 4. አልስማማም 5. በጣም አልስማማም |  |  |
| 357 | በተወሰነ ግዜ የደም ግፊትን መለካት አስፈላጊ ነዉ፡፡ | 1. በጣም እስማማለሁ 2. እስማማለሁ 3. እርግጠኛ አይደለሁም 4. አልስማማም 5. በጣም አልስማማም |  |  |
| 358 | የአካል ብቃት እንቅስቃሴ ማድረግ የደም ግፊት በሽታን ይከላከላል፡፡ | 1. በጣም እስማማለሁ 2. እስማማለሁ 3. እርግጠኛ አይደለሁም 4. አልስማማም 5. በጣም አልስማማም |  |  |
| 359 | የደም ግፊት ህሙማን ስብነት ያላቸዉን ምግቦች ቢመገቡ ጥሩ ነዉ፡፡ | 1. በጣም እስማማለሁ 2. እስማማለሁ 3. እርግጠኛ አይደለሁም 4. አልስማማም 5. በጣም አልስማማም |  |  |
| 360 | የደም ግፊት ህመም ያለባቸዉ ሰዎች ጭንቀት ከሚፈጥሩ ነገሮች መራቅ አለባቸዉ፡፡ | 1. በጣም እስማማለሁ 2. እስማማለሁ 3. እርግጠኛ አይደለሁም 4. አልስማማም 5. በጣም አልስማማም |  |  |
| 500 **መለኪያ** | | | |  |
| 501 | ክብደት | ________________ ኪ.ግ |  |  |
| 502 | ቁመት | ________________ |  |  |
| 503 | የ ወገብ ዙሪያ ስፋት (በሤ.ሜ) | ______________ሤ.ሜ |  |  |
| 504 | የዳሌ ዙሪያ ስፋት (በሤ.ሜ) | _______________ሤ.ሜ |  |  |
| 505 የደምግፊት ልኬት | | | |  |
|  | ልኬት 1 | ስይስቶሊክ________ዳያስቶሊክ________( በሚሊሜትርሜርኩሪ |  |  |
|  | ልኬት 2 | ስይስቶሊክ________ዳያስቶሊክ________በሚሊሜትርሜርኩሪ |  |  |

**ከህመመተኛው የህክምና መረጃ ቻርት የሚሞሉ መረጃዎች መጠይቅ**

ማስታወሻ፤ ከዚህ በታች በሰንጠረዡ የተጠቀሱት መጠይቆች ከህመመተኛው የህክምና መረጃ ቻርት በጥንቃቄ ሊሞሉ ይገባል፡፡

| ተ.ቁ | ጥያቄዎች | መልስ | ዝለል | ምርመራ |
| --- | --- | --- | --- | --- |
| **ከህክምናጋርየተያያዙ መጠይቆች** | | | | |
| 201 | የ አሁኑ የኤድስ በሽታ ደረጃ? | 1. አንደኛ ደረጃ 2. ሁለተኛ ደረጃ 3. ሶስተኛ ደረጃ 4. አራተኛ ደረጃ |  |  |
| 202 | ህክምና ሲጀምሩ የነበራቸው የነጭ የደም ህዋስ መጠን? | __________cells/mm3 |  |  |
| 203 | አሁን ያላቸው የነበራቸው የነጭ የደም ህዋስ መጠን? | __________cells/mm3 |  |  |
| 213 | በአሁን ወቅት እየወሰዱት ያለው የጸረ ኤች አይ ቪ መድሃኒት ምንድነው? | 1. ዚዶቩዲን/ ላሚቩዲን/ ኔቪራፒን 2. ዚዶቩዲን/ ላሚቩዲን/ ኢፋቪሬንዝ |  |  |
| 217 | እያንዳንዱን የ ጸረ ኤች አይ ቪ መድሃኒት ለምን ያህል ጊዜ ወሰዱ (በወራት)? | __________________ |  |  |
| 218 | አብዛኛውን ጊዜ የተጠቀሙት የ ጸረ ኤች አይ ቪ መድሃኒት ምንድነው? | 1. እሰታቩዲን/ላሚቩዲን/ ኔቪራፒን 2. እስታቩዲን/ ላሚቩዲን/ ኢፋቪሬንዝ 3. ዚዶቩዲን/ላሚቩዲን/   ኔቪራፒን   1. ዚዶቩዲን/ላሚቩዲን/ኢፋቪሬንዝ 2. ቴኖፎቪር/ላሚቩዲን/ ኔቪራፒን 3. ቴኖፎቪር/ላሚቩዲን/ ኢፋቪሬንዝ 4. ሌላ………………. |  |  |
| 221 | ተጓዳኝ የህመም አይነት አለ? | 1. አዎን____ 0.አይደለም___ |  |  |
| 222 | ለጥያቄ ቁጥር 221 መለሱ አዎን ከሆነ የተገኘው የተጓዳኝ የህመም አይነት ምንድነው? | 1. የ ቆዳ ኢንፌክሽን 2. ነቀርሳ 3. የ ጉበት ቫይረስ ቢ 4. የ ጉበት ቫይረስ ሲ 5. ሌላ ( ይጥቀሱ)--------- |  |  |
| 228 | የ አሁኑ የ ቫይረስ ክምችት መጠን ? | ____________ኮፒ በ ሚሊ. ሊትር |  |  |
| 229 | የ አሁኑ ከፍትኛ ዴንሲቲያለው የስብ መጠን? | ________________ሚ.ግ በ ዲ.ሊ |  |  |
| 230 | የ አሁኑ ዝቅተኛ ዴንሲቲያለው የስብ መጠን? | ________________ሚ.ግ በ ዲ.ሊ |  |  |
| ህክምና ከ መጀመራቸው በፊት የ ነበረ የልኬት ሰውነት ሁኔታ | | | | |
| 401 | ህክምና ከ መጀመራቸው በፊት የ ነበራቸው የሰውነት ክብደት  በ ኪ.ግ. | ___________ኪ.ግ |  |  |

የመረጃ ሰብሳቢው ስም_________________________________

ፊርማ__________________

ቀን___________________
