# Supplementary material for: The magnitude of hypertension and associated factors among clients on highly active antiretroviral treatment in Southern Ethiopia, 2020: A hospital-based cross-sectional study
Source: PLoS One. 2021 Oct 15;16(10):e0258576. doi: 10.1371/journal.pone.0258576 (PMC8519467; doi:10.1371/journal.pone.0258576)
Supplement: Attachment — Submitted filename: Reviewer comment_Saro.docx [file pone.0258576.s003.docx]

**Abstract**

Line 64 – Finish the sentence, it says “and contributing factors…….” contributing factors toward what? may be death, morbidity or what?. On the same line, please replace the phrase “as a result” with another.

Please re-write the result section in the abstract well. You may use a phrase like “the magnitude/proportion of hypertensive individuals among PLHIV on HAART was found to be -----. Associated factors with what? Please write explicitly.

Conclusion and recommendation should be inline with the findings. Line 87 “a concerted effort is required among health care providers to halt the habit of high alcohol use” is this the only intervention? Does your finding support this recommendation?

**Introduction**

Line 123 write PHA in words

Line 127 sentence that start with “As the available studies are very limited…….” You may replace it by “knowledge gap about…..” You better split the sentence in two. Change some phrases line “attracted attention” you may replace it by “neglected”

Line 129 and 130 – Is diagnosis of hypertension not a routine activity? BP is measured at every OPD, right?

Line 133 – Pease correct the grammar – “Besides, existing studies have since been completed”

Line 135 – “paid particular attention” please use standard words in study like “made clear, investigated, etc”

137-140 – a very long sentence, please break it down

# **Material and Methods**

Line 147 – what are the 4 major departments? On the same line, abbreviations shall be defined when first come in text – HMIS

Line 153 – Please rephrase as “All adult clients on HAART ……”

Line 165 – “a double population formula was employed……” do you have two populations? Is it a cohort study?

Line 168 - Hypertension in unexposed and exposed – what is exposur in this study? CD4, why?

Line 155-156 – “Adult clients receiving HAART and available within the data collection period were included in the study.” Line 180 – “Finally, after selecting the first client randomly, every 3^rd^ adult client was included and interviewed.” The two sentences contradict each other.

Line 156-157 – Please include reasons for exclusion of subjects and make sure the findings will not be biased as a result.

Line 191-193 – please reconsider rewriting, it is not coherent and wordy.

Line 198 –“ intensive” is a relative word and usually used to indicate higher/more quantity of something. One day or two days training or orientation? Can we call it intensive?

Line 272 – it seems that you only refer to those cases with extremely high or low BP, what is this extremely? You may change it to all hypertensive and hypotensive patients

# **Results**

Line 278 – capitalize “O” in over

Line 279/280 – “As well” is slung word

Line 293 - Under “Clinical Related Characteristics” sub section you can use “had ” instead of “were having”

In table 1 - Occupation – where did you categorized students and company/non-governmental organization employee?

Line 300-302 – “4.8% had >= 130mg/dl and the mean value 72.1 mg/dl.” The mean value can not be less than 130mg/dl.

Table 2. Some words in the title starts with capital letter and some with small letter. Pls keep it uniform

Table 2. - Majority 88% of participants had CD4 less than 200. Opportunistic infections are common with such CD4 level. You reported only less than half of the participants had history of OI. Please check again and give insight.

Line 309-312 – Instead of reporting weight for adults, it will be more meaningful if you report BMI

Line 324 – “per day per week” is not clear

Table 4 salt consumption – in a community that prepare their food for the whole family with salt and consumes together, how did you quantify individual salt intake?

Fig. 1 what is bad attitude towards hypertension prevention? Do they love to have hypertension?

Line 338-340 – coherence issue, you may put the second sentence in the first sentence position.

# **Discussion**

Line 368 – it says baseline BMI is significantly associated with hypertension, but table 5 shows baseline BMI was not associated with hypertension. Why you chose to include baseline BMI? If you want to see obesity and hypertension, then you should choose the current BMI.

404 and 405 – sentence repeated

409 – Wordy sentence

411 – Grammar not correct, please re write the sentence. ‘….health care provider to halt the habit of…..’ it is not the habit of health care provider. It is participants’ habit.

418 – What is “positively” please write the effect explicitly.

422-431 – this paragraph would give sense if you use current BMI. Otherwise, BMI before years may not be associated with current hypertension and lipid profile directly.

**Conclusion**

Refer 411 and 418 comments

Line 448- repeated words in a sentence study, study and again examine. Please make it concise.
