# Supplementary material for: The magnitude of hypertension and associated factors among clients on highly active antiretroviral treatment in Southern Ethiopia, 2020: A hospital-based cross-sectional study
Source: PLoS One. 2021 Oct 15;16(10):e0258576. doi: 10.1371/journal.pone.0258576 (PMC8519467; doi:10.1371/journal.pone.0258576)
Supplement: Attachment — Submitted filename: Manuscript to be reviewed.docx [file pone.0258576.s004.docx]

**The Magnitude of Hypertension and Associated Factors among Clients Receiving Highly Active Antiretroviral Treatment in Southern Ethiopia, 2020 (Hospital-Based Cross-Sectional Study)**

Kaleegziabher Lukas, ^1^ Endrias Markos, ^2^ Fanuel Belayneh, ^2^ Aklilu Habte, ^3*^

^1^Epidemiology Department, School of public health, College of Medicine and Health Sciences, Wachemo University, Hosanna, Southern Ethiopia

OOF

COPY

Not for

publication

ORIGINAL RESEARCH

Determinants of Drug-Resistant Tuberculosis in

Southern Ethiopia: A Case–Control Study

This article was published in the following Dove Press journal:

5Infection and Drug Resistance

Daniel Biru

1

Endrias Markos

Woldesemayat

2

1

UNICEF Ethiopia, Addis Ababa, Ethiopia;

2

Hawassa University, College of Medicine

and Health Sciences, School of Public

Health, Hawassa, Ethiopia

^2^Hawassa University, School of Public Health, College of Medicine and Health Sciences Hawassa, Ethiopia

^3^ Reproductive Health department, School of public health, College of Medicine and Health Sciences, Wachemo University, Hosanna, Southern Ethiopia

*Corresponding Author: E-mail: [**akliluhabte57@gmail.com**](mailto:akliluhabte57@gmail.com)

# **ABSTRACT**

**Introduction**

Following the introduction of Highly Active Anti Retro Viral Treatment (HAART), the survival of people living with HIV/AIDS (PLHIV) has improved. However, hypertension remains a major challenge for people living with HIV. Very little effort has been made to examine hypertension and contributing factors in clients receiving HAART, particularly in southern Ethiopia. As a result, this study aims to determine the extent of hypertension and associated factors in clients receiving HAART at Wachemo University Nigist Eleni Mohammed Memorial Referral Hospital, southern Ethiopia, 2020.

**Methods**:

A hospital-based cross-sectional study took place between January 20 and March 20, 2020. A systematic sampling technique was employed in the selection of 382 clients. Interviewer administered pretested structured questionnaire was used for data collection. Blood pressure and anthropometric parameters of PLHIV were measured. The data was encoded and entered using Epi Data Version 3.1 and exported to SPSS version 23 for analysis. Then bivariable and multivariable logistic regression analyses were used to identify associated factors. Adjusted Odds Ratio (AOR) with 95% CI was used to present the estimated effect size and declare the presence of statistically significant association respectively.

**Results**:

The magnitude of hypertension in the study setting was 11.0% 95% CI [7.93,14.04]. Being on HAART for at least 60 months (AOR: 2.57, 95% CI: 1.24-5.21), being on TDF/3TC/EFV combination (AOR: 4.61, 95% CI: 2.52-8.3), and high alcohol drinking (AOR: 4.31, 95% CI: 1.84-10.02) were independent factors found to have an association with hypertension among clients receiving HAART.

**Conclusion and Recommendation**:

The magnitude of hypertension in the study setting was in a considerable state to plan and implement intervention measures. A due Emphasis should be placed on planning interventions to reduce hypertension in clients who have received TDF/3TC/EFV and TDF/3TC/NVP and those who have received HAART for ≥60 months. Moreover, a concerted effort is required among health care providers to halt the habit of high alcohol use through counseling and education to reduce the occurrence of hypertension.

**Keywords**: Hypertension, HAART, PLHIV, Blood Pressure, Ethiopia

# **Introduction**

The human immunodeficiency virus (HIV), a very common communicable disease, remains one of the major public health challenges[1]. By the end of 2018, about 37.9 million people worldwide were living with HIV, with sub-Saharan Africa being the most affected region, with 25.6 million people living with HIV in the region, sharing two-thirds of all cases worldwide [2]. In Ethiopia, 722,248 people were living with HIV, of which approximately 23,000 were newly infected by annual AIDS-related deaths of 11,000, and prevalence and incidence were 0.4% and 1% respectively[3]. Acquired Immune Deficiency Syndrome (AIDS) has become central to many global concerns and has reached epidemic proportions in some parts of the world[4].

Hypertension is an elevated systemic blood pressure condition that is typically asymptomatic. It is a major cardiovascular risk factor that is closely associated with severe complications such as coronary artery disease, stroke, heart failure, and kidney failure[5]. HIV can cause inflammation in the blood vessels, boost atherosclerosis, and the formation of high-risk plaques, which increases the risk of cardiovascular diseases[6]. In addition, some antiretroviral medications used to treat HIV also increased cholesterol levels, abdominal fat, and blood pressure[7].

While, since the beginning of HARRT has made great progress in improving the longevity and quality of life of PLWHIV, treatment is not risk-free or side effects. Complications such as dyslipidemia and related high blood pressure are the most commonly encountered complications of HAART, and it has a well-established increased risk of cardiovascular morbidity in people infected with HIV[8-11]. In HIV-positive people who receive HAART, death results from cardiovascular complications such as hypertension related to the virus, host and ART factors[9-11].

Hypertension in HIV-infected adults is linked to a higher incidence of persistent proteinuria, coronary heart disease, and myocardial infarction than in subjects not infected with HIV[12]. Of all the comorbidities in HIV-positive populations, hypertension appeared among the main causes of AIDS-unrelated mortality[13]. The prevalence of hypertension among people living with HIV is 4.7-54.4% in high-income countries and 8.7-45.9% in low- and middle-income countries[14].

Studies in people infected with HIV have reported various metabolic changes associated with HAART, including alterations in lipid and glucose metabolism, peripheral arterial disease, and coronary artery disease. Moreover, the simultaneous occurrence of hypertension and HIV among people infected with HIV can complicate the management of HIV infection, increasing the risk of morbidity and mortality of these people[15, 16].

Although the burden of non-communicable diseases, especially hypertension in PHA, is significant, few studies have demonstrated the negative impact of ART, suggesting the presence of NCDs and contributing factors. To date, in Ethiopia, very limited attempts have been made to study the magnitude of hypertension and contributing factors in clients receiving HAART[11, 17]. As the available studies are very limited and the co-morbidity of HIV and NCDs among clients on antiretroviral therapy is not well addressed, solutions for preventing hypertension have not attracted attention. To date, the diagnosis, prevention, and management of hypertension are not routine tasks and have not focused on risk reduction among clients receiving HAART. It is therefore believed that this study helps determine the magnitude of hypertension and the factors contributing to the situation.

Besides, existing studies have since been completed that did not address other relevant factors such as lipodystrophy, knowledge, and attitude regarding the prevention of hypertension [11, 17, 18]. Also, this study paid particular attention to the features related to ART, including the current ART regimen, regimen change, the most commonly used ART regimen, adherence to HAART, and opportunistic infection. As such, this research will help to provide a clear description of the magnitude of hypertension and its determinants in clients receiving HAART in Nigist Elleni Mohammed Memorial Referral Hospital which is one of the biggest HAART centers in the southern region.

# Material and Methods

## **Study design and setting**

A hospital-based cross-sectional study was conducted at Wachemo University Nigist Elleni Mohammed Memorial Referral Hospital (NEMMRH) from January 20 to March 20, 2020. The hospital is located 232 km from Addis Ababa, the capital of Ethiopia, and 157 km from Hawassa, the capital of the southern region. NEMMH under Wachemo University has 201 beds giving services in the 4 major departments. According to information obtained from the hospital's HMIS office, the hospital began delivering the HAART service in 2006 GC. It provides services with 4 general practitioners, 3 health officers, 3 nurses, and 3 pharmacists who have been trained in ART. Now it is among the largest ART centers in southern Ethiopia serving over 1,100 adult clients receiving HAART.

## **Populations of the study**

## All adult HAART clients visiting WCUNEMMRH during the data collection period were the source population. Selected clients aged 18 and over and who met all inclusion criteria during the data collection period were the study population. Adult clients receiving HAART and available within the data collection period were included in the study. Patients who received corticosteroids, women who were confirmed pregnant and took hormonal contraceptives for more than six months, and seriously ill patients during data collection were excluded. **Sample size determination**

The sample size for the first objective was determined by using the single population proportion formula. By using the following parameters: the estimated proportion of hypertension among clients receiving HAART in Eastern Ethiopia was 12.7% [11], the margin of error 3% since the prevalence is small, 95% level of confidence, and 10% non-response rate, the ultimate sample size for the study were 519. Correction formula is applied since the population is less than 10,000 and hence the final sample size for the first objective was 352. Secondly, a double population formula was employed with consideration of factors associated with hypertension among clients on HAART. Among those factors selected, the largest sample size(n=620) was obtained by considering percent of hypertension in unexposed(i.e. current CD4 count <500cells/mm3=90%), AOR of 2.7 [11]. Besides other parameters like; 80% power, 95% confidence level, 5% degree of precision, the ratio of unexposed to exposed equally to 1, non-response rate of 10%. Again correction formula is applied and the final sample size for the second objective was 397. Finally, the sample size obtained using two population proportion considerations (n=397) was larger than the sample size for single population proportion (n=352) and it was taken as the final sample size for the study.

## **Sampling techniques**

A systematic sampling technique was employed to include study participants. Subsequently, K was determined after dividing the total enrolled clients by the sample size (i.e. 1200/397=3). Finally, after selecting the first client randomly, every 3^rd^ adult client was included and interviewed.

## **Data collection tools, methods, and personnel**

Structured questionnaires were developed after an in-depth review of the literature on the area of interest[11, 16, 19, 20]. The questionnaire comprised of multiple segments: socio-demographic, behavioral, clinical, and anthropometric factors. WHO standardized tool was used to assess behavioral and lifestyle factors. Stress was assessed using Cohen’s 10 items Perceived Stress Scale (PSS) [21]. Diet-related questions were assessed using a Guideline, Dietary Approach to Stop Hypertension (DASH). Secondary data like WHO staging, baseline CD4 cell count, base line-height and weight, baseline viral load were extracted from client medical records. Data were collected by a face-to-face interview on exit, by two trained BSc nurses with the supervision of two public health officers. Both data collectors and supervisors were provided with intense training that lasts out two days regarding the point towards the study, ways, and data collection techniques by going all through the questionnaire one by one.

## **Data Quality management**

The questionnaire used to collect the data was first prepared in English and translated into the local language by an expert in that language, then translated into English to ensure consistency with the original meanings. a pre-test was carried out in 5% of the sample size (20 clients) at the Worabe Comprehensive Specialized Hospital. Data collectors and supervisors received intensive one-day training. The data collected has been verified for completeness and consistency on the date of data collection. Every day, the principal investigator and supervisor supervised the overall data collection. To mitigate a biased estimation weight scale was calibrated to zero before each measurement and tested with a standard scale.

**Definition and operationalization of variables of the study**

BP was measured based on the new WHO HEARTS recommendation [19], where, the participant was in sitting position and his/her back being supported, leg uncrossed, empty bladder, not talking and the patient has taken an adequate rest for 5 minutes and was measured by 2 BSc Nurses using a mercury sphygmomanometer and stethoscope having adult size cuff. The BP measurement was consistently taken 2 times from the left arm 5 minutes apart and the second reading was taken to be estimated and used in the analysis. Hypertension was classified as a BP measure of Systolic ≥ 140 mmHg and /or/ Diastolic ≥ 90 mmHg[19].

All anthropometric measurements were taken twice and the average was taken. The weight of the participants was taken using the standard beam balance and the scale was verified and calibrated at zero before each measurement. Participants’ weight was measured after removing heavy clothing and recorded to the nearest 0.1KG. Height measurement of participants was taken using the standard measuring scale. Participants’ take off their shoes, stand erect, in a horizontal plane. The occiput, shoulder, buttocks, and heels touched the measuring board, and height was recorded to the nearest 0.1cm. Waist circumference was measured at the midpoint between the lowest margin of the last palpable rib and the top iliac crust while the patient is breathing and standing and was categorized according to the WHO standards. Hip circumference was measured at the point yielding the maximum circumference over the buttocks with the tape in a horizontal plane, touching but not compressing the skin. Waist to Hip Ratio(WHR) was calculated as mean waist circumference divided by mean hip circumference and to interpret the result WHO cutoff was used for males and females [11].

Cigarette Smoker: A study participant is considered a cigarette smoker if he/she smokes now or has smoked cigarettes in the past, daily or less than daily [22].

Family history of NCDs: A study participant was considered to have a family history of NCD if he had a history of NCD with his siblings, mother, or father.
Good physical activity for Health: A study participants were considered to be having a good physical activity if they had at least 150 minutes of moderate physical activity or at least 75 minutes of vigorous physical activity during work, Transportation, and leisure all week long[23].

Good knowledge about prevention of hypertension: A study participant was considered to have a good knowledge of hypertension prevention if he or she scored above the average of the 10 knowledge questions[24].

alcohol drinking: considered as no/mild, moderate, and high alcohol consumer if he/she scored < 8, 9-14 and scored more than 15 of the questions on alcohol consumption respectively[25].

Stress level: A study participants should be considered as low, medium, and high if they scored 0-13, 14-26, and 27-40 on 40 Perceived Stress Scale (PSES) questions, respectively[21].

History of Treatment to Chronic Diseases: if he/she has a history of treatment for either of the following conditions; cardiovascular diseases, TB, Hypertension Kidney Diseases, and DM.

Khat Chewer: One study participant who chews khat even once within 30 days before the study was considered a khat chewer.

Lipodystrophy: A study participant with fat loss in the face or arms and/or fat gain in the back or at the base of the neck, increased breast size, decreased fat on the buttocks or legs will be considered lipodystrophy.

A negative attitude about prevention of hypertension: if he/she scored lower than the average of the 8 attitude questions. [24].

Adherence to HAARTs-: A study participant was considered as adhered to HAART if he /she didn’t miss even a single dose of the prescribed regimens in the past one week[26].

Regimen Change: A study participant was considered as having a history of regimen change if he/she switched or substitute for at least one drug from the original HAART regimen for the first time due to several reasons.

## **Data analysis**

After verifying its completeness and consistency, the data was entered using EpiData version 3.1 and exported to SPSS version 23 for analysis. Descriptive statistics such as frequency distributions, mean, median standard deviation, and inter-quartile range have been calculated to quantify the variables. Bivariable and multivariable logistic regression was used to identify factors associated with hypertension. In the bivariable analysis, each explanatory variable was tested for the presence of an association with the outcome variable and the significant variables at P <0.25 were candidates for multivariable logistic regression. Lastly, independent variables with P <0.05 in multivariable logistic regression were reported as statistically significant variables with the dependent variable. The adjusted odds ratio (AOR) and with it 95% CI were used to report the association.

## **Ethical approval and consent to participate**

Ethical clearance was obtained from the Institutional Review Board of Hawassa University College of Medicine and Health Sciences and a support letter was written to NEMMRH officials and concerned bodies about the purpose and importance of the study. Written informed consent for the research was given by all participants. Respondents were also well informed that the information provided during the study would only be used for research purposes and would not be disclosed to anyone other than the research team. To keep the questionnaire anonymous, a unique identification number was issued. Confidentiality and privacy were preserved throughout all the study procedures. Additionally, participants with extremely high and low BP during the study were linked to the relevant care provider and were trying to obtain appropriate care.

# **Results**

## **Socio-demographic characteristics of respondents**

Of the total 397sampled study participants, 382 have participated in the study which yielded a response rate of 96.2%. Respondents were aged 19-63, with the median age being 35 (IQR: 29-43), and the majority (60.8%) were aged 31 to 50. over one-half (53.9%) of respondents were women. Concerning educational attainment, 138 (36.1%) had completed secondary school. As well, 245 (64.1%) and 259 (67.8%) were Protestant and belonged to the Hadiya ethnic group, respectively (Table1).

Table 1: Socio-demographic characteristics of clients receiving HAART at Nigist Elleni Mohammed Memorial Referral Hospital (NEMMRH), Southern Ethiopia, January 20 –March 20, 2020.

| **Variables** | **Frequency** | **Percentage** |
| --- | --- | --- |
| **Age in years(n=382)** |  |  |
| 19-30 | 114 | 29.8 |
| 31-50 | 232 | 60.8 |
| >=51 | 36 | 9.4 |
| **Sex(n=382)** |  |  |
| Male | 176 | 46.1 |
| Female | 206 | 53.9 |
| **Religion(n=382)** |  |  |
| Protestant | 245 | 64.1 |
| Orthodox | 66 | 17.3 |
| Muslim | 65 | 16.9 |
| Catholic | 6 | 1.5 |
| **Ethnicity(n=382)** |  |  |
| Hadiya | 259 | 67.8 |
| Kembata | 89 | 23.2 |
| Amhara | 34 | 8.9 |
| **Educational Status(n=382)** |  |  |
| No formal education | 80 | 20.8 |
| Primary education | 129 | 33.7 |
| Secondary education | 138 | 36.1 |
| College and above | 36 | 9.4 |
| **Marital Status(n=382)** |  |  |
| Married | 157 | 41.1 |
| Single | 120 | 31.4 |
| Divorced | 47 | 12.3 |
| Widowed | 58 | 15.2 |
| **Occupation(n=382)** |  |  |
| Housewife | 119 | 31.2 |
| Farmer | 58 | 15.2 |
| Merchant | 55 | 14.4 |
| Daily laborer | 82 | 21.4 |
| Civil Servant | 68 | 17.8 |
| **Residence(n=382)** |  |  |
| Urban | 250 | 65.4 |
| Rural | 132 | 34.6 |
| **Average Monthly Income(n=382)** |  |  |
| <=500ETB | 164 | 43 |
| 501-1500ETB | 146 | 38.2 |
| >=1501 ETB | 72 | 18.8 |

## **Clinical Related Characteristics**

Close to nine in ten respondents (88.2%) had a reference CD4^+^ cell count of <200 cells/mm3 and 357 (93.5%) had a recent CD4^+^ cell count of >= 200 cells/mm3 or higher to date. A recent viral load of 50–1000 copies/ml was recorded for 335 (87.7%) of participants. Lypodystrophy was observed for 86 clients (22.5%). Two hundred fifty-two (66.5%) had a history of regimen change. Whereas, 120(30.2%) were currently on AZT/3TC/EFV. During the preceding 7 days, 283(74.1%) reported that they had treatment interaction. Two hundred ninety-five (92.7%) were having an HDL level of >= 40mg/dl, with a mean of 63(SD± 10) mg/dl. High LDL level i.e. >= 130mg/dl was apparent among 15(4.8%) of participants with the mean (±Standard deviation S/D) of 72.1(±10.5) mg/dl. As well, 300 PLWHIV (78.5%) reported having already measured their BP. Also, 164(42.9%) were having a history of opportunistic infection and of these, 101(61.5%) were having skin disorders (Table 2).

Table 2: Clinical Related characteristics of clients Receiving HAART at NEMMRH, Southern Ethiopia, January 20-March 20, 2020.

| **Clinical characteristics of respondents** | **Frequency** | **Percentage** |
| --- | --- | --- |
| **Baseline CD_4_^+^ cell count(n=382)** |  |  |
| < 200 cells/ mm^3^ | 337 | 88.2 |
| >= 200 cells/ mm^3^ | 45 | 11.8 |
| **Recent CD_4_^+^ cell Count(n=382)** |  |  |
| <500 cells/ mm^3^ | 25 | 6.5 |
| >=500 cells/ mm^3^ | 357 | 93.5 |
| **Duration of HAART(n=382)** |  |  |
| >= 60 months | 137 | 35.9 |
| < 60 months | 245 | 64.1 |
| **Lipodystrophy(n=382)** |  |  |
| Yes | 86 | 22.5 |
| No | 296 | 77.5 |
| **History of Opportunistic Infection(n=382)** |  |  |
| No | 218 | 57.1 |
| Yes | 164 | 42.9 |
| **Family History of NCDs(n=382)** |  |  |
| Yes | 54 | 14.1 |
| Don't know | 52 | 13.6 |
| No | 276 | 72.3 |
| **Regimen Change(n=382)** |  |  |
| Yes | 252 | 65.9 |
| No | 130 | 34.1 |
| **Current HAART Regimen(n=382)** |  |  |
| TDF/3TC/EFV | 68 | 17.8 |
| TDF/3TC/NVP | 94 | 24.6 |
| AZT/3TC/EFV | 120 | 31.4 |
| AZT/3TC/NVP | 100 | 26.2 |
| **Adherence to HAART(n=382)** |  |  |
| Yes | 283 | 74.1 |
| No | 99 | 25.9 |
| **Recent Viral Load(n=382)** |  |  |
| 50-1000 copies/ml | 335 | 87.7 |
| 1001-10000copies/ml | 47 | 12.3 |
| **HDL (n=318)** |  |  |
| <40mg/dl | 23 | 7.3 |
| >=40 mg/dl | 295 | 92.7 |
| **LDL** **(n=318)** |  |  |
| >=130mg/dl | 15 | 4.8 |
| <130 mg/dl | 303 | 95.2 |

## **Anthropometric measurement**

The mean **(**± SD) for the baseline weight of participants was 42.7 (± 6.3) Kg, whereas, the current weight being 56 (± 4.2) Kg. The mean (±SD) height was 1.6 (± 0.11) M. Three hundred twelve (81.6%) were underweight at the start of their HAART. Men subjects having a high WHR were 13(3.4%) (Table 3).

Table 3. Anthropometric Related Characteristics of respondents receiving HAART at NEMMRH, Southern Ethiopia, January 20-March 20, 2020.

| **Anthropometric variables (n=382)** | **Frequency** | **Percentage** |
| --- | --- | --- |
| **Baseline BMI** |  |  |
| <18.5 Kg/M² | 312 | 81.7 |
| 18.5-24.9 Kg/M² | 65 | 17.0 |
| >=25Kg/M² | 5 | 1.3 |
| **Current BMI** |  |  |
| <18.5Kg/M² | 65 | 17.0 |
| 18.5-24.9Kg/M² | 313 | 82.0 |
| >=25Kg/M² | 4 | 1.0 |
| **Male Waist to Hip Ratio** |  |  |
| >= 0.9 | 13 | 7.3 |
| < 0.9 | 163 | 92.7 |
| **Female Waist to Hip Ratio** |  |  |
| >= 0.85 | 3 | 1.4 |
| < 0.85 | 203 | 98.6 |

## **Behavioral Characteristics of Participants**

A good level of physical activity was reported by 200(52.4%) of respondents. Three hundred sixty-one (94.5%) were nonsmokers and for the item asking about alcohol consumption frequency, 266 (69.6%) replied that they had a habit of low-level alcohol drinking in the past one year. More than seven in ten (71.7%) had no prior history of chewing khat and 228 (59.7%) were with low-stress levels. Regarding the dietary practice, 235(59.3%) reported that they had a vegetable intake of 1-7 times per day per week (Table4).

Table 4: Diet Related characteristics of clients receiving HAART at NEMMRH, Southern Ethiopia, January 20-March 20, 2020.

| **Variables(n=382)** | **Frequency** | **Percentage** |
| --- | --- | --- |
| **Fruit Intake** |  |  |
| 1-7 times/week | 183 | 47.9 |
| None | 199 | 52.1 |
| **Vegetable Intake** |  |  |
| 1-7 times | 234 | 61.3 |
| None | 148 | 38.7 |
| **Type of Oil used to prepare a meal** |  |  |
| Saturated | 230 | 60.2 |
| Unsaturated | 152 | 39.8 |
| **Salt Consumption** |  |  |
| High | 281 | 73.6 |
| Low | 101 | 26.4 |

High salt Consumption: ≥1 teaspoon; ≥2300mg of Na/day.

Low salt Consumption : <1 teaspoon; ≤2300mg of Na/day.

## **Knowledge and attitude towards Hypertension prevention measures**

The questions assessing knowledge about preventing hypertension were passed along to the study participants and 236 (61.8%) had good knowledge about preventing hypertension. Besides, 128 (33.5%) had a negative attitude toward preventing hypertension (Figure1).

**Fig1. Participants' knowledge and attitude on prevention of hypertension of receiving HAART at NEMMRH, Southern Ethiopia, January 20-March 20, 2020.**

## **The magnitude of hypertension among clients receiving HAART**

In this study, hypertension was diagnosed based on the new WHO HEARTS protocol. Of a total of 382 participants, 42 (11.0%) had hypertension. The mean systolic and diastolic blood pressure of participants was 108 mmHg (SD±15) and 78mmHg (SD±10) respectively. Thirty (7.8%) were having a systolic BP measure of ≥140mmHg and 12(3.2%) were having a diastolic BP measure of ≥90mmHg (Figure2).

Fig2. The magnitude of hypertension among clients receiving HAART at NEMMRH from January 20-March 20, 2020.

## **Factors associated with hypertension among clients receiving HAART**

A multivariable logistic regression analysis was carried out and four variables namely; duration of staying on HAART, baseline BMI, high alcohol consumption, and the current HAART regimen were significantly associated with being hypertensive. This study has shown that staying on HAART for a long time increases the likelihood of developing hypertension. Clients who had been taking HAART for ≥60 months were 2.57 times more likely to develop hypertension than their counterparts (AOR: 2.57, 95% CI: 1.24-5.21). Besides, those who consumed high levels of alcohol were 4.3 times more likely to develop hypertension than those who consumed low levels (AOR: 4.31, 95% CI: 1.84-10.02). It was also determined that the type of current regimen taken by clients showed a significant association with hypertension. Those, who were on TDF/3TC/EFV combinations had 4.6 times increased odds of developing hypertension when compared to those on AZT/3TC/NVP (AOR: 4.61, 95% CI: 2.52-8.3) (Table5)

Table 5 Bivariable and Multivariable Logistic Regression analysis of factors associated with hypertension among clients receiving HAART at NEMMRH from January 20-March 20, 2020.

| **Respondents’ characteristics** | **Hypertension** | | **COR(95%CI)** | **AOR(95%CI)** |
| --- | --- | --- | --- | --- |
|  | **Yes (%)** | **No (%)** |  |  |
| **Age group** |  |  |  |  |
| 18-30 | 2(5.7) | 33(94.3) | 1 | 1 |
| 31-50 | 13(5.9) | 205(94.1) | 1.046(0.1,2.19) | 0.423(0.08,2.06) |
| >= 51 | 27(20.9) | 102(79.1) | 4.36(0.5,2.06) | 0.742(0.33,1.62) |
| **Sex** |  |  |  |  |
| Female | 26(12.7) | 179(87.3) | 1.45(0.75,2.80) | 1.54(0.741,3.20) |
| Male | 16(9.1) | 161(90.9) | 1 | 1 |
| **Baseline BMI** |  |  |  |  |
| <18.5 Kg/ M² | 6(9.7) | 56(90.3) | 1 | 1 |
| >= 18.5 Kg/ M² | 36(11.2) | 284(88.7) | 2.64(0.90,4.80) | 1.75(0.75,3.35) |
| **Duration of HAART** |  |  |  |  |
| >= 60 months | 22(16.1) | 115(83.9) | 2.15(1.0,4.01)* | 2.57(1.24,5.2) ** |
| < 60 months | 20(8.2) | 225(91.8) | 1 | 1 |
| **Regimen Change** |  |  |  |  |
| Yes | 32(12.7) | 220(87.3) | 1.70(0.80,3.58)* | 1.27(0.55,2.89) |
| No | 10(7.7) | 120(92.3) | 1 |  |
| **Current HAART Regimen** |  |  |  |  |
| TDF/3TC/EFV | 14(20.6) | 54(79.4) | 3.44(1.30,9.06)* | 4.61(2.52,8.3)** |
| TDF/3TC/NVP | 15(15.9) | 79(84.1) | 2.52(0.98,6.49)* | 2.36(1.7,5.8)** |
| AZT/3TC/EFV | 6(5.0) | 114(95.0) | 0.69(0.22,1.2) | 1.4(0.40,4.79) |
| AZT/3TC/NVP | 7(7.0) | 93(93.0) | 1 | 1 |
| **Alcohol Drinking** |  |  |  |  |
| High | 19(16.4) | 97(83.6) | 2.06(1.07,3.97)* | 4.3(1.84,10.2)** |
| Low | 23(8.7) | 243(91.3) | 1 | 1 |
| **Waist to Hip Ratio** |  |  |  |  |
| >= 0.85 | 2(28.6) | 5(71.4) | 3.33(0.62, 17.7)* | 0.38(0.05,2.81) |
| <0.85 | 40(10.7) | 335(89.3) | 1 | 1 |
| **Fruit Intake** |  |  |  |  |
| 1-7/week | 27(13.6) | 172(86.4) | 1.75(0.90,3.42)* | 1.62(0.74,3.53) |
| None | 15(8.2) | 168(91.8) | 1 | 1 |

* Those Variables statistically significant at p-value <0.25 in bivariable analyses.

** Those Variables statistically significant at p-value <0.05 in multivariable analyses.

# **Discussion**

This transversal hospital study was attempted to assess the extent of hypertension and its associated factors in HAART clients. The magnitude of hypertension in the study setup was 11.0 %. The factors identified significantly associated with hypertension were, being on TDF/3TC/EFV and TDF/3TC/NVP combinations, high alcohol consumption, baseline BMI, and being on HAART for 60 months and more.
 the magnitude of hypertension in this study was consistent with the findings from Kenya and Eastern Ethiopia in which 13.4% and 12.7% of clients receiving HAART were hypertensive[11, 27]. While the magnitude is lower than studies carried out in the USA (31.0%), Nigeria (40.9%), Uganda (24.8%), southern Ethiopia (15.9%), and southwestern Ethiopia (16.0%)[16-18, 28, 29]. The difference can be attributed to the age differences of participants in which 61% of respondents in this study were aged 31-50 years, while the age group was higher(>=50yrs) in those studies with higher magnitude, this, in turn, raises the risk of hypertension[17, 18, 28, 29]. In addition, the hyperlipidemia status in this study was 12%, which is lower than previous studies done elsewhere[16, 29], which can lead to increased levels of lipids in blood vessels, which in turn increases the risk of hypertension[4, 30]. Furthermore, the type of HAART taken by clients may be the potential cause of the variance. The majority of respondents to the current study are taking HAART which are nucleoside reverse transcriptase (NRTI) inhibitors, while clients of other studies with high magnitude were taking protease inhibitors (IP), these drugs are known to raise lipid levels and thus cause hypertension[29, 31].

This study found that taking HAART over a long period increases the likelihood of developing hypertension. Clients who had been on HAART for ≥60 months were more likely to develop hypertension than their counterparts. This is consistent with studies carried out elsewhere in Ethiopia[11, 18]. This can be explained by the fact that clients taking HAART for a long time may develop abdominal fat and cholesterol, which can facilitate the onset of hypertension by changing the normal physiological conditions of the body[7]. In addition, some HAART facilitates the development of high blood pressure, due to the mechanism of change in lipid and glucose metabolism resulting in an increase in peripheral and coronary heart disease over time[4, 15]. This finding contradicted a study in southern Ethiopia that found no link between the duration of ART and hypertension[17]. This discrepancy could be due to the threshold differences used for the duration of HAART, in the current study it was ≥60 months, but in the previous study, it was ≥ 4 years. Furthermore, the discrepancy can be explained by differences in physical activity and work intensity. In the current study, the proportion of participants with good physical activity was 52.4%, versus 69% in the previous one. As a result, it is essential to implement intervention measures and focus on clients who are taking HAART to reduce the occurrence of hypertension.

The present study has shown that high alcohol intake is associated with hypertension. Those who drank a lot of alcohol were 4.3 times more likely to develop hypertension than those who drank little. This finding was supported by a similar study done in Eastern Ethiopia[11]. Alcohol increases BP, through its adverse effect on blood vessels that paves the way for hypertension. The results of this study contradict studies in Uganda and Tanzania which found no link between alcohol use and hypertension[28, 32]. This disparity may be explained by the type of tool used to determine the level of alcohol consumed. This discrepancy can be explained by the type of tool used to determine the level of alcohol consumed, this study used the WHO tool for alcohol consumption and disorders. The level of knowledge of the participants may also account for the difference. Under the present study, a significant number of participants had poor knowledge about preventing hypertension which could lead to them not being able to be restrained and protect themselves from what is harmful to their health and this will expose them to hypertension. Therefore, a concerted effort is required among the unit's health care providers to halt the habit of high alcohol use through counselling and education must be considered in order to reduce hypertension among PLWHIV.

In this study, the type of HAART regimen currently used increased the risk of hypertension. In particular, respondents who took the TDF/3TC/EFV and TDF/3TC/NVP combination were 4.6 and 2.3 times more likely to develop hypertension than those clients with AZT/3TC/NVP. This was supported by a similar study conducted in Cameroon, in which the regimens, TDF/3TC/EFV and TDF/3TC/NVP were positively associated with hypertension[20]. This may be due to the nature of protease inhibitor(IP) regimens which are known to increase lipid levels and therefore cause hypertension[29, 31]. Therefore, it is essential to plan strategies to reduce hypertension in clients who receive those selected HAART regimens.

Unlike many studies in Senegal, Malawi, Tanzania, and southern Ethiopia[11, 32-34], baseline BMI is not associated with hypertension. This may be because, in this study, about 82% of study participants had a baseline BMI of <18.5Kg/m2 at the initiation of HAART. Moreover, most of the study participants were underweight when they began HART in this study. In contrast, a higher proportion of participants were obese in previous studies, 61.1% and 25%, respectively, in Tanzania and Malawi[32, 34], and just 2.5% in the current study. Another possible rationale for the lack of association in the current study could be a lower state of hyperlipidemia (4.7%) compared to a similar study in Tanzania (61.3%)[32] since dyslipidemia facilitates the development of atherosclerosis and plaque formation in blood vessels predisposing to hypertension[4, 8].

Unlike other studies, the current study has sought to address factors that have not been addressed in other studies such as recent viral load, opportunistic infections, regimen change, adherence with HAART, and knowledge and attitudes on preventing hypertension. Although considerable efforts have been made to minimize the bias effect in this study, it will not be exempt from the recall bias. Moreover, because of the nature of the study as a cross-sectional study cannot elucidate the cause-effect relationship. Lastly, the study was based on self-reporting, which could have resulted in a social desirability bias.

# **Conclusion**

Hypertension among clients receiving HAART in this study was in a significant state for the planning and implementation of intervention measures. Factors positively associated with hypertension in this study were: being on HAART for ≥60 months, high alcohol consumption, being on TDF/3TC/EFV and TDF/3TC/NVP combinations. Emphasis should be placed on planning interventions to reduce hypertension in clients who have received TDF/3TC/EFV and TDF/3TC/NVP and clients who have received HAART for ≥60 months. Therefore, a concerted effort is required among health care providers to halt the habit of high alcohol use through counseling and education must be considered to reduce the occurrence of hypertension among PLWHIV. Other prospective studies are recommended to study other predictors of hypertension and to examine the clear effects of the current HAART regimen on hypertension.

**Supporting information**

S1 data (Data set)

S2 data (Questionnaire for CPNC)

**List of abbreviations**

**AIDS**: Acquired Immuno Deficiency Syndrome; **AOR**: Adjusted Odds Ratio; **ATV**: Atazanavir; **AZT**: Zidovudine; **BMI**: Body Mass Index; **D4T**: Stavudine; **EFV**: Efavirenz; **HAART**: Highly Active Anti Retro Viral Treatment; **NEMMRH**; Nigist Elleni Mohammed Memorial Referral Hospital; **NVP**: Nevirapine; **PLWHIV**: People Living With HIV; **SPSS**: Statistical product and service solutions; **WHO**: World Health Organization

**Acknowledgments**

We are indebted to Hawassa University College of Medicine and Health Sciences School of Public Health for giving Ethical clearance to undertake the study. Our appreciation also goes to the managers and healthcare providers who worked in Nigist Elleni Mohamed Memorial Referral Hospital for their assistance and cooperation during the study. Finally, for their efforts, we want to thank our supervisors, data collectors, and study participants.

**Funding statement**

## There was no funding to report

**Availability of data and materials**

The data used to strengthen the results of this study are fully available without restriction

**Competing interest:**

We would like to assure you that, with full responsibility and communication, the document has been submitted. We indicated that there is no conflict of interest with other individuals or organizations that could influence or bias the content of the paper inappropriately.

# References

1.	Sherwood, J., et al., *HIV/AIDS National Strategic Plans of Sub-Saharan African countries: an analysis for gender equality and sex-disaggregated HIV targets.* Health policy and planning, 2017. **32**(10): p. 1361-1367.

2.	Unaids, J., *Fact sheet—latest global and regional statistics on the status of the AIDS epidemic.* Geneva: UNAIDS, 2017.

3.	Kibret, G.D., et al., *Trends and spatial distributions of HIV prevalence in Ethiopia.* Infectious diseases of poverty, 2019. **8**(1): p. 90.

4.	Loscalzo, J., et al., *Harrison's Principles of Internal Medicine: Self-Assessment and Board Review*. 2017: McGraw-Hill Professional.

5.	Longo, D.L., et al., *Harrison's principles of internal medicine.* NEW YORK: McGraw-Hill CO, 2012. **6**(1): p. 312.

6.	Eugenin, E.A., et al., *Human immunodeficiency virus (HIV) infects human arterial smooth muscle cells in vivo and in vitro: implications for the pathogenesis of HIV-mediated vascular disease.* The American journal of pathology, 2008. **172**(4): p. 1100-1111.

7.	Krishnan, S., et al., *Changes in metabolic syndrome status after initiation of antiretroviral therapy.* Journal of acquired immune deficiency syndromes (1999), 2015. **68**(1): p. 73.

8.	Grover, S.A., et al., *Impact of dyslipidemia associated with Highly Active Antiretroviral Therapy (HAART) on cardiovascular risk and life expectancy.* The American journal of cardiology, 2005. **95**(5): p. 586-591.

9.	Shahmanesh, M., et al., *Protease inhibitor related type III hyperlipoproteinaemia is common and not associated with apolipoprotein-E E2/E2 phenotype.* Sexually transmitted infections, 2001. **77**(4): p. 283-286.

10.	Rhee, M.S., et al., *Relationship Between Plasma Protease Inhibitor Concentrations and Lipid Elevations in HIV Patients on a Double‐Boosted Protease Inhibitor Regimen (Saquinavir/Lopinavir/Ritonavir).* The Journal of Clinical Pharmacology, 2010. **50**(4): p. 392-400.

11.	Ataro, Z., et al., *Magnitude and associated factors of diabetes mellitus and hypertension among adult HIV-positive individuals receiving highly active antiretroviral therapy at Jugal Hospital, Harar, Ethiopia.* HIV/AIDS (Auckland, NZ), 2018. **10**: p. 181.

12.	Jung, O., et al., *Hypertension in HIV-1-infected patients and its impact on renal and cardiovascular integrity.* Nephrology Dialysis Transplantation, 2004. **19**(9): p. 2250-2258.

13.	Sackoff, J.E., et al., *Causes of death among persons with AIDS in the era of highly active antiretroviral therapy: New York City.* Annals of internal medicine, 2006. **145**(6): p. 397-406.

14.	Nguyen, K.A., et al., *Burden, determinants, and pharmacological management of hypertension in HIV-positive patients and populations: a systematic narrative review.* AIDS Rev, 2015. **17**(2): p. 83-95.

15.	Peck, R.N., et al., *Hypertension, kidney disease, HIV and antiretroviral therapy among Tanzanian adults: a cross-sectional study.* BMC medicine, 2014. **12**(1): p. 125.

16.	Medina-Torne, S., et al., *Hypertension is common among HIV-infected persons, but not associated with HAART.* Journal of the International Association of Physicians in AIDS Care, 2012. **11**(1): p. 20-25.

17.	Sachithananthan, V., E. Loha, and M. Gose, *Prevalence of diabetes mellitus, hypertension and lipodystrophy in HAART receiving HIV patients in Southern Ethiopia.* International STD Research & Reviews, 2013: p. 1-11.

18.	Berhane, T., et al., *Prevalence of lipodystrophy and metabolic syndrome among HIV positive individuals on Highly Active Anti-Retroviral treatment in Jimma, South West Ethiopia.* Pan African Medical Journal, 2012. **13**(1).

19.	Organization, W.H., *Technical package for cardiovascular disease management in primary health care: evidence-based treatment protocols*. 2018, World Health Organization.

20.	Pangmekeh, P.J., et al., *Association between highly active antiretroviral therapy (HAART) and hypertension in persons living with HIV/AIDS at the Bamenda regional hospital, Cameroon.* The Pan African Medical Journal, 2019. **33**.

21.	Tesfaye, S.H. and G.T. Bune, *Generalized psychological distress among HIV-infected patients enrolled in antiretroviral treatment in Dilla University Hospital, Gedeo zone, Ethiopia.* Global health action, 2014. **7**(1): p. 23882.

22.	Organization, W.H. and C.f.D. Control, *Tobacco questions for surveys: a subset of key questions from the Global Adult Tobacco Survey (GATS): global tobacco surveillance system.* 2011.

23.	Organization, W.H., *Global physical activity questionnaire (GPAQ) analysis guide*. 2012, Geneva.

24.	Aschalew, A. and T. Dube, *Knowledge of Hypertension among Ethiopian Army to the African Peace Keeping Mission.* Epidemiology (Sunnyvale), 2019. **9**(369): p. 2161-1165.1000369.

25.	Organization, W.H., *AUDIT: The alcohol use disorders identification test: Guidelines for use in primary health care*. 2001, Geneva: World Health Organization.

26.	Tiyou, A., et al., *Predictors of adherence to antiretroviral therapy among people living with HIV/AIDS in resource-limited setting of southwest ethiopia.* AIDS Research and Therapy, 2010. **7**(1): p. 39.

27.	Manuthu, E., et al., *Prevalence of dyslipidemia and dysglycaemia in HIV infected patients.* East African medical journal, 2008. **85**(1): p. 10-17.

28.	Mateen, F.J., et al., *Hypertension prevalence and Framingham risk score stratification in a large HIV-positive cohort in Uganda.* Journal of hypertension, 2013. **31**(7): p. 1372-1378.

29.	Denue, B.A., et al., *Effects of highly active antiretroviral therapy (HAART) on blood pressure changes and its associated factors in HAART naive HIV-infected patients in north eastern Nigeria.* Archives of Applied Science Research, 2012. **4**(3): p. 1447-1452.

30.	Fontas, E., et al., *Lipid profiles in HIV-infected patients receiving combination antiretroviral therapy: are different antiretroviral drugs associated with different lipid profiles?* The Journal of infectious diseases, 2004. **189**(6): p. 1056-1074.

31.	Palacios, R., et al., *Impact of highly active antiretroviral therapy on blood pressure in HIV‐infected patients. A prospective study in a cohort of naive patients.* HIV medicine, 2006. **7**(1): p. 10-15.

32.	Kagaruki, G.B., et al., *Magnitude and risk factors of non-communicable diseases among people living with HIV in Tanzania: a cross sectional study from Mbeya and Dar es Salaam regions.* BMC public health, 2014. **14**(1): p. 904.

33.	Benzekri, N.A., et al., *Increasing prevalence of hypertension among HIV-positive and negative adults in Senegal, West Africa, 1994-2015.* PloS one, 2018. **13**(12): p. e0208635.

34.	Muronya, W., et al., *Cardiovascular risk factors in adult Malawians on long-term antiretroviral therapy.* Transactions of the Royal Society of Tropical Medicine and Hygiene, 2011. **105**(11): p. 644-649.
