# Supplementary material for: The magnitude of hypertension and associated factors among clients on highly active antiretroviral treatment in Southern Ethiopia, 2020: A hospital-based cross-sectional study
Source: PLoS One. 2021 Oct 15;16(10):e0258576. doi: 10.1371/journal.pone.0258576 (PMC8519467; doi:10.1371/journal.pone.0258576)
Supplement: Attachment — Submitted filename: Response to Reviewers.docx [file pone.0258576.s005.docx]

**AUTHORS’ RESPONSE TO ACADEMIC EDITOR**

**Dear: Academic Editor**

We thank you for a thorough reading and constructive criticism of our manuscript and for the opportunity to revise and resubmit. We are pleased to submit the revised research article “**The Magnitude of Hypertension and Associated Factors among Clients Receiving Highly Active Antiretroviral Treatment in Southern Ethiopia, 2020: A Hospital-Based Cross-Sectional Study**” for your consideration in the special collection of Plos One. On the following pages, you will find our response to the editor’s comments. On behalf of my co-authors, I thank you for your consideration of this resubmission. We appreciate your time and look forward to your response.

Sincerely,

Aklilu Habte(MPH)(corresponding author)

[**aklilihabte57@gmail.com**](mailto:aklilihabte57@gmail.com)

**Journal Requirements:**

**Comment:** Please ensure that your manuscript meets PLOS ONE's style requirements, including those for file naming. The PLOS ONE style templates can be found at <https://journals.plos.org/plosone/s/file?id=wjVg/PLOSOne_formatting_sample_main_body.pdf> and<https://journals.plos.org/>plosone/s/file?id=ba62/PLOSOne_ formatting_sample_title_ authors_affiliations.pdf

**Response:** Thank you for your suggestion, and we do so according to the requirements of the journal.

**Commen2:** In statistical methods, please refer to any post-hoc corrections to correct for multiple comparisons during your statistical analyses. If these were not performed please justify the reasons. Please refer to our statistical reporting guidelines for assistance (<https://journals.plos.org/plosone/s/submission-guidelines.#loc-statistical-reporting>).

**Response:**

**Comment3:** We suggest you thoroughly copyedit your manuscript for language usage, spelling, and grammar. If you do not know anyone who can help you do this, you may wish to consider employing a professional scientific editing service.

**Response:** Thank you for your suggestion and now, in the current submission, we have given due emphasis to language, spelling and grammar errors by consulting language experts at our university and all the corrected items have been highlighted in the "Revised Manuscript with Track Changes”

**Response to Additional Editor Comments:**

**Comment1:** dear Authors follow reviewer suggestions to improve your paper

**Response:** Thank you for your invitation to respond to the comments and suggestions made by the esteemed reviewers, and we do so by responding point-by-point to each reviewer's suggestion.

__________________________________________________________________________________________________________END________________________________________

THANK YOU!!!

**AUTHORS’ RESPONSE TO REVIEWERS**

**DEAR: REVIEWERS**

We thank you for a thorough reading and constructive criticism of our manuscript and for the opportunity to revise and resubmit. We are pleased to submit the improved research article “The Magnitude of Hypertension and Associated Factors among Clients Receiving Highly Active Antiretroviral Treatment in Southern Ethiopia, 2020 : A Hospital-Based Cross-Sectional Study” for your consideration in the special collection of Plos One. On the following pages, you will find our response to the each reviewers’ comments. On behalf of my co-authors, I thank you for your consideration of this resubmission. We appreciate your time and look forward to your response independently as for reviewer 1 and 2 respectively.

Sincerely,

Aklilu H. (MPH) (corresponding author)

[**aklilihabte57@gmail.com**](mailto:aklilihabte57@gmail.com)

**AUTHORS’ RESPONSE TO REVIEWER**S

**1. Is the manuscript technically sound, and do the data support the conclusions?**

The manuscript must describe a technically sound piece of scientific research with data that supports the conclusions. Experiments must have been conducted rigorously, with appropriate controls, replication, and sample sizes. The conclusions must be drawn appropriately based on the data presented.

Reviewer #1: Partly

Reviewer #2: Yes

**Response:** We thank the reviewer for kind words regarding the soundness of our study.

2. Has the statistical analysis been performed appropriately and rigorously?

Reviewer #1: I don’t know

Reviewer #2: no

**Response:** Thank you for your input on the statistical analysis aspect of our study. In the "data analysis" portion of the "revised manuscript with track changes" section, we will try to explain the statistical part more clearly.

3. Have the authors made all data underlying the findings in their manuscript fully available?
The [PLOS Data policy](http://www.plosone.org/static/policies.action#sharing) requires authors to make all data underlying the findings described in their manuscript fully available without restriction, with rare exception (please refer to the Data Availability Statement in the manuscript PDF file). The data should be provided as part of the manuscript or its supporting information, or deposited to a public repository. For example, in addition to summary statistics, the data points behind means, medians and variance measures should be available. If there are restrictions on publicly sharing data—e.g. participant privacy or use of data from a third party—those must be specified.

Reviewer #1: Yes

Reviewer #2: Yes

**Response:** Thank you for ensuring the availability of all the relevant data.

4. Is the manuscript presented in an intelligible fashion and written in standard English?
PLOS ONE does not copyedit accepted manuscripts, so the language in submitted articles must be clear, correct, and unambiguous. Any typographical or grammatical errors should be corrected at revision, so please note any specific errors here.

Reviewer #1: Yes

Reviewer #2: No

Thank you for the comment raised by the reviewer 2 and we have given due emphasis to language, spelling and grammar errors by consulting language experts at our university and all the corrected items have been highlighted in the "Revised Manuscript with Track Changes”

**AUTHORS’ RESPONSE TO REVIEWER** #**1**

**Comment1:** the objectives of the study are not clear and should be stated in the initial study design section

**Response:** Thank you very much for your meticulous review and now, in the "Abstract" portion of the "revised manuscript with track changes," Page2, Line65-66, we have tried to clarify the purpose of the study. We also tried to mention the objectives of the study.In the "Introduction" section, and highlighted on Page5, Line147-149 of the “revised manuscript with track changes”

**Comment2**: the sample size calculation section is confusing. It should be clear what the primary objective is and the assumptions used to calculate a sample size to meet that primary objective.

**Response:** We appreciate your thorough review of our manuscript. To make it clearer, we have been using sample size calculation for both the first and the second objectives to get the maximum sample size. Our primary objective was to determine the magnitude of hypertension among clients receiving HAART, and for this section, we applied the single population proportion formula. Our second objective was to determine the contributing factors among those clients for Hypertension and to estimate the sample size, we used a double population proportion formula by considering all significant variables in prior studies. The variable "CD4 count" provided the maximum sample size of these selected variables for sample size calculation. Finally, we took the maximum sample size for the study after comparing the two sample sizes.

Regarding the “hypertension among exposed and unexposed”, these are the parameters during sample size determination by using Statcalc Menu of Epi Info Version7 and taken from the related literature.

While it is not widely used in prior research, most scholars at the national level currently suggest determining the sample size for both first and second objectives to achieve the optimal sample size. During the proposal process of the current work, scholars at the college level have suggested us to do so.

**Comment3:** The sampling strategy is also unclear. Were the clients listed randomly and then every third chosen? Were they stratified in any way? Or just every third chosen from the existing client register? This needs to be clarified in the manuscript.

**Response:** We appreciate and accept your point of view on the sampling procedure and now we have tried to make it clear and all the improvements have been highlighted in the "sampling techniques" section of the “revised manuscript with track changes”, on Page 6, Line 192-194.

**Comment4:** Please address why no participants appear to be on second line PI-based regimens

**Response:** we appreciate your commnet. according to the current HAART guideline, second line –PI regimens were recommended during severe adverse drug reaction and During document review accordingly couldn’t get any client with such complaint.

**Comment5:** The frequency of hyperlipidemia is listed at 12 percent but it is not clear how this is defined or measured. Medical history or self-report is unlikely to be accurate unless there is comprehensive screening for hyperlipidemia as a standard of care in this setting.

**Response:** Thank you for your comments, and we share all of your concerns as well. In the "Data collection tools, techniques and personnel" section of the "method" portion, we tried to mention that secondary data such as WHO staging, baseline CD4 cell count, base line-height and weight, baseline viral load, HDL level, LDL level, and Opportunistic Infection were extracted from client medical records. And now we have kindly assured you that figures related to hyperlipidemia were originated from the medical record of the client.

**Comment6:** It is not clear how the alternate measurement tool could account for the alcohol use finding. This should be addressed more specifically

**Response:** Thank you for the comment and our approach to the evaluation of behavioral factors was based on self-report, and by including additional results from the evaluation, we tried to narrate the result part. The measurement of alcohol consumption was available in the data collection tool section and referred to as "S1-questionnaire" in the "supporting information" files.

**Comment7:** The authors should discuss potential explanations for TDF based regimens being a risk factor and the implications of the TLD transition

Response: Thank you for your suggestion and we have attempted to mention the possible justifications for the association in the “discussion” section of the “revised manuscript with track changes”, on Page19, Line480-482

**Comment8:** I don't think use of contraceptives or receipt of corticosteroids are appropriate exclusion criteria. It would be important to know how common these exclusions were.

**Response:** We value your point of view. But the majority of studies on hypertension exclude those clients who use contraceptives. Hormonal contraceptives like Depo-Provera take the line-share for contraceptive adoption in the current study setup, and their side effect is hypertension. So we exclude them because of the fear of over-estimating the outcome of interes. Regarding the corticosteroid, we did not get those respondents even set it in the exclusion criteria and we omit it and highlighted on Page6, Line172 the “revised manuscript with track changes”’

**Minor comments**

**Comment9:** probably more appropriate to use the term "frequency" of hypertension rather than "magnitude"

**Response:** Thank you for your comment and we corrected it in the revised manuscript as per your suggestion.

**Comment10:** Prefer "renal failure" to "kidney failure"

**Response:** thank you for your comment and we correct it as per your suggestion and highlighted it on Page3, Line107 of the “revised manuscript with track changes”

**Comment11:** prefer "people living with HIV" to "HIV-positive people"

**Response:** we got your suggestion as a better way and we amend it accordingly and highlighted it in the “revised manuscript with track changes”, Page3&4, Line115 and 120

**Comment12:** Recommend consistently use ART instead of HAART

**Response:** As per your recommendation, we amend it

**Comment13:** It would be helpful to describe the usual practice of blood pressure screening for HIV clinic visits at the hospital where participants were recruited and the usual standard of care for hypertension management in this setting.

**Response:** Thank you for your suggestion. We have tried to elaborate the procedure of blood pressure screening in the "Data collection tool, methods, and personnel" section of the “Revised manuscript with track changes”, On Page7, Line201-206

**Comment14:** It would be acceptable to just cite the methods for hypertension definition as opposed to detailing in the manuscript

**Response:** We welcome your painstaking examination of the manuscript. As per your suggestion, we move the detailed BP measurement procedure from the "measurement of variables of the study" section to the "Data collection tools, methods and personnel" section of the "revised manuscript with track changes", On Page7, Line201-206. The definition of hypertension is highlighted in the “measurement of variables of the study” section of the “revised manuscript with track changes”, on Page8, Line247-248.

**Comment15:** Could consider using a separate table to list out the many definitions

**Response:** Thanks for the suggestion you made. But we assumed that these variables are not many to consider to present separate tables. As per your suggestion, we have attempted to reduce the unnecessary procedural statements previously stated in the "variable of the study" section by moving them to the "Data collection tools, methods, and personnel" section of the revised manuscript.

**Comment16:** describe what a recent viral load is considered to be in the paper (within the past year?)

**Response:** Thank you for your detailed review, and we'd like to say sorry for that confusing term. We used the term "recent" just to denote the "current viral load" and "current CD4 count" showing the results during the last test and we have now corrected and highlighted it in table 2 of the “revised manuscript with track changes”, on Page 12 and 13

**Comment17:** What is the "reference" CD4? The nadir or baseline? Recommend clarification.

**Response:** Thank you for the concern that has been posed. In the current study, the baseline CD4 corresponds to the CD4 count at the initiation of HAART.

__________________________________________________________________________________________________________END________________________________________

THANK YOU!!!

**AUTHOR’S RESPONSE TO REVIEWER**#**2**

**Abstract**

**Comment1:** Line 64 – Finish the sentence, it says “and contributing factors…….” contributing factors toward what? May be death, morbidity or what?. On the same line, please replace the phrase “as a result” with another.

**Response:** Thank you for the comment. Just we accept the comment and it is now replaced by “the magnitude of hypertension and its contributing factors” and highlighted on Page2, Line64 of the “revised manuscript with track change”

We also replaced the word “as a result” by “hence”, on Page2, Line65 of the “revised manuscript with track change”

**Comment2:** Please re-write the result section in the abstract well. You may use a phrase like “the magnitude/proportion of hypertensive individuals among PLHIV on HAART was found to be -----. Associated factors with what? Please write explicitly.

**Response:** thank you for kind suggestion and we amend the result section of the abstract as per your recommendation and highlighted on Page2, Line78-82 of the “revised manuscript with track change”

**Comment3:** Conclusion and recommendation should be in line with the findings. Line 87 “a concerted effort is required among health care providers to halt the habit of high alcohol use” is this the only intervention? Does your finding support this recommendation?

**Response:** Thanks for your reviews. Based on the results, we gave two recommendations and these are: 1) For those clients who have received TDF/3TC/EFV and TDF/3TC/NVP and those who have been on HAART for 60 months, a strong emphasis should be placed on planning a strict follow-up. 2)Also, a concerted effort is required by health care providers to discourage the habit of high alcohol consumption through counseling and education. All these recommendations were highlighted on Page2-3, Line 86-89 of the “revised manuscript with track change”. Yes! Yes! Our conclusion and recommendations, of course, were drawn from the results of the present study.

**Comments from the “Introduction” section**

Comment4: Line 123 write PHA in words

**Response:** Thank you for the comment and we correct it as “People living with HIV” and highlighted it on Page4, Line129 of the “revised manuscript with track change”

**Comment5:** Line 127 sentence that start with “As the available studies are very limited…….” You may replace it by “knowledge gap about…..” You better split the sentence in two. Change some phrases line “attracted attention” you may replace it by “neglected”

**Response:** We appreciate your meticulous review and we earn it as a valuable contribution to the readability of our work from your suggestion. We have amended the sentence according to your recommendation and highlighted it on Page4, Line133-134 of the “revised manuscript with track change”

**Comment6:** Line 129 and 130 – Is diagnosis of hypertension not a routine activity? BP is measured at every OPD, right?

**Response**: Thank you for having raised your concerns. The measurement of blood pressure is one of the daily activities at health facilities, but the applicability varies from one point of service delivery to the other. The HAART regimen was the main focus of health care providers, especially in HAART clinics, and less attention was given to other routine activities like BP measurement.

**Comment7:** Line 133 – Pease correct the grammar – “Besides, existing studies have since been completed”

**Response:** As per your suggestion, we corrected the sentence and highlighted it on Page 4, Line 140-141 of the “revised manuscript with track change”.

**Comment8:** Line 135 – “paid particular attention” please use standard words in study like “made clear, investigated, etc”

**Response:** we amend the statement and the corrected one is highlighted on Page4, Line144-146 of the “revised manuscript with track change”

**Comment9:** 137-140 – a very long sentence, please break it down

**Response:** Thank you for the suggestion. The lengthy statement is due to the hospital's long name, " Nigist Elleni Mohammed Memorial Referral Hospital”. We try to shorten the sentence, however, and the corrected one is highlighted on Page4, Line 147-149 of the “revised manuscript with track change”

# Material and Methods

**Comment10:** Line 147 – what are the 4 major departments? On the same line, abbreviations shall be defined when first come in text – HMIS

**Response:** Ok, thanks for reminding us. We include the names of the departments that are the departments of surgery, gynecology and obstetrics, internal medicine, and pediatrics highlighted on Page5, Line159-160 of the “revised manuscript with track changes”. The Abbreviation “HMIS” denoted for “Health Management Information System” and now we write it in text and highlighted it on Page5, Line160-161 of the “revised manuscript with track changes”

**Commnet11:** Line 153 – Please rephrase as “All adult clients on HAART ……”

**Response:** thank you for the comment and we rephrased the statement as per your suggestion and highlighted it in the “Population of the study” section of the “revised manuscript with track changes”, Page6, Line 166-168.

**Commnet12:**Line 165 – “a double population formula was employed……” do you have two populations? Is it a cohort study? , Hypertension in unexposed and exposed – what is exposur in this study? CD4, why?

**Response:** We appreciate your thorough review of our manuscript. To make it clearer, we have been using sample size calculation for both the first and the second objectives to get the maximum sample size. Our primary objective was to determine the magnitude of hypertension among clients receiving HAART, and for this section, we applied the single population proportion formula. Our second objective was to determine the contributing factors among those clients for Hypertension and to estimate the sample size, we used a double population proportion formula by considering all significant variables in prior studies. The variable "CD4 count" provided the maximum sample size of these selected variables for sample size calculation. Finally, we took the maximum sample size for the study after comparing the two sample sizes.

Regarding the “hypertension among exposed and unexposed”, these are the parameters during sample size determination by using Statcalc Menu of Epi Info Version7 and taken from the related literature.

While it is not widely used in prior research, most scholars at the national level currently suggest determining the sample size for both first and second objectives to achieve the optimal sample size. During the proposal process of the current work, scholars at the college level have suggested us to do so.

**Commnet13:** Line 155-156 – “Adult clients receiving HAART and available within the data collection period were included in the study.” Line 180 – “Finally, after selecting the first client randomly, every 3^rd^ adult client was included and interviewed.” The two sentences contradict each other.

**Response:** thank you for your meticulous review and now we correct the first statement as “Adult clients on HAART were included in the study” in the “Population of the study” section of the “revised manuscript with track changes, on Page 6, Line170-171. We have rewritten the “sampling techniques” section again and highlighted it on Page7, Line192-195 of the “revised manuscript with track changes”.

**Commnet14:** Line 156-157 – Please include reasons for exclusion of subjects and make sure the findings will not be biased as a result.

Response: We value your point of view. But the majority of studies on hypertension exclude those clients who use contraceptives. Using Hormonal contraceptives like Depo-Provera take the lion-share for contraceptive uptake in Ethiopia includingthe current study setup, and hypertension is found to be one of their common side effect. So we exclude them because of the fear of over-estimating the result. Regarding the corticosteroid, we did not get those respondents even we set it in the exclusion criteria and we omit it in the “revised manuscript with track changes”, Page6, Line172

**Commnet15:** Line 191-193 – please reconsider rewriting, it is not coherent and wordy.

Response: Thank you for showing the way to revise the sentence and we got it as a redundant idea, and now we have removed it from the manuscript and highlighted it on Page8, Line 227-229 of the “revised manuscript with track changes”.

**Commnet16:** Line 198 –“ intensive” is a relative word and usually used to indicate higher/more quantity of something. One day or two days training or orientation? Can we call it intensive?

**Response:** Thank you for your suggestion. Many publications used the word intensive training and we removed it as per your suggestion and write simply as “a one-day training”

**Comment17:** Line 272 – it seems that you only refer to those cases with extremely high or low BP, what is this extremely? You may change it to all hypertensive and hypotensive patients

**Response:** Thank you for your suggestion, and we've corrected it accordingly. The amended statement is highlighted on Page10, Line 307-309 of the “revised manuscript with track changes”.

# Results

**Comment18:** Line 278 – capitalize “O” in over

**Response:** corrected and highlighted on Page11, Line316of the “revised manuscript with track changes”.

**Comment19:** Line 279/280 – “As well” is slung word

**Response:** corrected and highlighted on Page11, Line317 of the “revised manuscript with track changes”.

**Commnet20:** Line 293 - Under “Clinical Related Characteristics” sub section you can use “had ” instead of “were having”

**Response:** Thank you for your suggestion and we have corrected it accordingly on Page 12, Line 330 of the “revised manuscript with track changes”.

**Comment21:** In table 1 - Occupation – where did you categorized students and company/non-governmental organization employee?

**Response:** We appreciate the question, but during the study period we could not access such respondents and here we reported what we got during data collection.

**Commnet22:** Line 300-302 – “4.8% had >= 130mg/dl and the mean value 72.1 mg/dl.” The mean value can not be less than 130mg/dl.

**Response:** Thank you for your thorough review, but we removed it, so it was unnecessary.

**Comment23:** Table 2. Some words in the title starts with capital letter and some with small letter. Pls keep it uniform

**Response:** As per your suggestion, we have corrected it accordingly and highlighted it on Page12, Line 337-338 of the “revised manuscript with track changes”.

**Comment24:**Table 2. - Majority 88% of participants had CD4 less than 200. Opportunistic infections are common with such CD4 level. You reported only less than half of the participants had history of OI. Please check again and give insight.

**Response:** We appreciate your meticulous review of our work. For clarification, the source of data about OIs was from the medical record of the client and the health care providers give less emphasis to document for each OIs than the CD4 result and this could be the reason behind this huge variation. The corrected statement is highlighted in on Page12, Line334-335 of the “revised manuscript with track changes”.

**Comment25:**Line 309-312 – Instead of reporting weight for adults, it will be more meaningful if you report BMI

**Response:** Thank you for your comment, but the BMI was also reported and highlighted on Page13, Line342-343, of the “revised manuscript with track changes”.

**Comment26:**Line 324 – “per day per week” is not clear

**Response:** Sorry for the error that we have committed and it was just to mean “per week”. We have corrected and highlighted it on Page 14, Line355 of the “revised manuscript with track changes”.

**Commnet27:** Table 4 salt consumption – in a community that prepare their food for the whole family with salt and consumes together, how did you quantify individual salt intake?

**Response:** our Initial intention was to assess the pattern of salt consumption at the house hold level. But now we have convinced with your idea that individual-level salt consumption could not be assessed based on self-report and we remove this from the manuscript and highlighted it on Page15, Line370-374 of the “revised manuscript with track changes”.

**Comment29:** Fig. 1 what is bad attitude towards hypertension prevention? Do they love to have hypertension?

**Response:** Thanks for the commnet. Initially, by using the Likert scale questions, the attitude of respondents towards Hypertension prevention was assessed and finally, we dichotomized it into “good/positive” and “poor/negative”. We aim to categorize the level of attitude of respondents just in the way that different literature has done. Now we replaced the word “bad attitude” with “poor attitude”.

**Commnet30:** Line 338-340 – coherence issue, you may put the second sentence in the first sentence position.

**Response:** Thank you for the suggestion, and we're doing it straight.

# Discussion

Commnet31: Line 368 – it says baseline BMI is significantly associated with hypertension, but table 5 shows baseline BMI was not associated with hypertension. Why did you choose to include baseline BMI? If you want to see obesity and hypertension, then you should choose the current BMI.

**Response:** We appreciate your meticulous review. We apologize for this inconvenience we made, and we're erasing it from the manuscript and we highlighted on Page16, Line415 of the “revised manuscript with track changes”.

**Commnet32:** 404 and 405 – sentence repeated

**Response:** we have avoided the redundant statement and highlighted it on Page18, Line451-452 of the “revised manuscript with track changes”.

**Comment33:** 409 – Wordy sentence

**Response:** Thank you to the commnet for that. We tried to make the statement more concise and more understandable, and highlighted on Page18, Line458-459 of the “revised manuscript with track changes”.

**Commnet34:** 418 – What is “positively” please write the effect explicitly.

**Response:** just to say “significantly” and we corrected and highlighted it on Page18, Line467 of the “revised manuscript with track changes”.

**Commnet33:** 422-431 – this paragraph would give sense if you use current BMI. Otherwise, BMI before years may not be associated with current hypertension and lipid profile directly.

**Response:** thank you for your suggestion and we share your idea with out any doubt. But almost all of the previous studies used Base line BMI and our comparison with the base line BMI of the respondents is due to that.

**Conclusion**

**Comment34:** Refer 411 and 418 comments

**Response:**Thank you for the commnet and we replaced the word “positively” by “significantly” and highlighted it on Page19, Line490 of the “revised manuscript with track changes”.

**Commnet35:** Line 448- repeated words in a sentence study, study and again examine. Please make it concise.

**Response:** as per your suggestion we tried to make the statement more concise and highlighted it on Page20, Line498-499 of the “revised manuscript with track changes”.

__________________________________________________________________________________________________________END________________________________________

THANK YOU!!!
