# Supplementary material for: The magnitude of hypertension and associated factors among clients on highly active antiretroviral treatment in Southern Ethiopia, 2020: A hospital-based cross-sectional study
Source: PLoS One. 2021 Oct 15;16(10):e0258576. doi: 10.1371/journal.pone.0258576 (PMC8519467; doi:10.1371/journal.pone.0258576)
Supplement: Attachment — Submitted filename: Response to Reviewers.docx [file pone.0258576.s006.docx]

**AUTHORS’ RESPONSE TO ACADEMIC EDITOR**

**Dear: Academic Editor(Professor Kwasi Torpey, MD PhD MPH)**

We thank you for a thorough reading and constructive criticism of our manuscript and for the opportunity to revise and resubmit. We are pleased to submit the revised research article “**The Magnitude of Hypertension and Associated Factors among Clients Receiving Highly Active Antiretroviral Treatment in Southern Ethiopia, 2020: A Hospital-Based Cross-Sectional Study**” for your consideration in the special collection of Plos One. On the following pages, you will find our response to the editor’s comments. On behalf of my co-authors, I thank you for your consideration of this resubmission. We appreciate your time and look forward to your response.

Sincerely,

Aklilu Habte(MPH)(corresponding author)

[**aklilihabte57@gmail.com**](mailto:aklilihabte57@gmail.com)

PONE-D-20-38405R1

The Magnitude of Hypertension and Associated Factors among Clients on Highly Active Antiretroviral Treatment in Southern Ethiopia, 2020: A Hospital-Based Cross-sectional Study

PLOS ONE

Dear Aklilu Habte,

Thank you for submitting your manuscript to PLOS ONE. After careful consideration, we feel that it has merit but does not fully meet PLOS ONE’s publication criteria as it currently stands. Therefore, we invite you to submit a revised version of the manuscript that addresses the points raised during the review process.

Please submit your revised manuscript by 15th Sept 2021. If you will need more time than this to complete your revisions, please reply to this message or contact the journal office at plosone@plos.org. When you're ready to submit your revision, log on to https://www.editorialmanager.com/pone/ and select the 'Submissions Needing Revision' folder to locate your manuscript file.

Please include the following items when submitting your revised manuscript:

A rebuttal letter that responds to each point raised by the academic editor and reviewer(s). You should upload this letter as a separate file labeled 'Response to Reviewers'.

A marked-up copy of your manuscript that highlights changes made to the original version. You should upload this as a separate file labeled 'Revised Manuscript with Track Changes'.

An unmarked version of your revised paper without tracked changes. You should upload this as a separate file labeled 'Manuscript'.

If you would like to make changes to your financial disclosure, please include your updated statement in your cover letter. Guidelines for resubmitting your figure files are available below the reviewer comments at the end of this letter.

If applicable, we recommend that you deposit your laboratory protocols in protocols.io to enhance the reproducibility of your results. Protocols.io assigns your protocol its own identifier (DOI) so that it can be cited independently in the future. For instructions see: http://journals.plos.org/plosone/s/submission-guidelines#loc-laboratory-protocols. Additionally, PLOS ONE offers an option for publishing peer-reviewed Lab Protocol articles, which describe protocols hosted on protocols.io. Read more information on sharing protocols at https://plos.org/protocols?utm_medium=editorial-email&utm_source=authorletters&utm_campaign=protocols.

We look forward to receiving your revised manuscript.

Kind regards,

Professor Kwasi Torpey, MD PhD MPH

Academic Editor

PLOS ONE

**Response:** We appreciate your detailed reading of our manuscript and insightful comments and suggestions, as well as the opportunity to review and resubmit it. On the following sections, we have attempted to respond to all of the comments made by the editor.

**Journal Requirements:**

Please review your reference list to ensure that it is complete and correct. If you have cited papers that have been retracted, please include the rationale for doing so in the manuscript text, or remove these references and replace them with relevant current references. Any changes to the reference list should be mentioned in the rebuttal letter that accompanies your revised manuscript. If you need to cite a retracted article, indicate the article’s retracted status in the References list and also include a citation and full reference for the retraction notice.

**Response:** Response: Thank you for recommending that we have checked the appropriateness of the references we used in the manuscript. We reviewed all of the references as per your suggestion, and none of them have retracted references.

**Response to Additional Editor Comments**

**Commen1:** There is a difference between the manuscript with tracked changes and the clean version. The tracked changes version captures most of the proposed changes. I suggest the authors to use the tracked changes version to generate the clean version paying attention to version control. Given the differences, the tracked changes version was used in this review

**Response:** Thank you for taking the time to examine the revised manuscript and the accompanying files that were attached to it. We'd like to let you know that the clean copy of the revised manuscript has all of the highlighted changes from the "revised manuscript with track changes". All of the improvements were emphasized in the current proposal based on your advice.

**Comment2:** Abstract/Results: Correct typo hypertensiion to hypertension

**Response:** Thank you for your detailed investigation; this has been corrected and marked in the "Abstract" section of the "Revised Manuscript with Track Changes", Page2, Line79

**Comment3:** Introduction page 3 last but one paragraph: Should be ART not ARRT

**Response:** thank you for the detailed review and we have corrected and highlighted it in the “Introduction” of the "Revised Manuscript with Track Changes", Page3, Line106

**Comment4:** last but one paragraph: Should be people living with HIV not people livening with HIV

**Response:** thank you for insightful reading of our work and we have corrected and highlighted it accordingly in the “Introduction” of the "Revised Manuscript with Track Changes", Page3, Line107

**Comment5:** Into last paragraph AIDS unrelated mortality should be non-AIDS related mortality

**Response:** thank you for the comment and we have corrected it accordingly and this was highlighted in the “Introduction” of the "Revised Manuscript with Track Changes", Page3, Line116

**Comment6:** Materials and methods: need to be consistent on the use of capitalization. For example, naming the departments ie, Some are capitalized others are not

**Response:** We appreciate your thorough evaluation, and we've made the necessary corrections, which are now highlighted in the "Materials and methods" section of the "Revised Manuscript with Track Changes", Page 4, Line 145.

**Comment7:** Population of the study line 172: remove comma and start capitalize w in women

**Response:** We have corrected and highlighted it in the “Population of the study” section of the "Revised Manuscript with Track Changes", Page5, Line 153

**Comment8:** Data quality management Line 236/7: Revise sentence. Data collected was verified for completeness. PI and supervisor oversaw overall data collection. Remove every day from sentence

**Response:** We appreciate your thorough review of our work, and we've made the changes you suggested in the data quality portion of the manuscript, which was highlighted in the "Revised Manuscript with Track Changes", Page7, Line218-220

**Comment9:** Conclusion: Revise opening statement . Seems out of place

**Response:** thank you for your suggestion and we have corrected the statement as per your suggestion and this was highlighted in the “Conclusion” section of the "Revised Manuscript with Track Changes", Page18, Line435-436.

**Comment10:** Manuscript needs another round of copyediting. Lipodystrophy instead of lypodystrophy. Keep spelling consistent

**Response:** Thank you for your thoughtful recommendation, and we have copyedited the document including the aforementioned word, with all corrected words highlighted throughout the revised manuscripts.

Comment11: Revise all references to meet journal requirements

Response: Thank you for your suggestion; we checked all of the references and found that they all met the journal's requirements.

__________________________________________________________________________________________________________END________________________________________

THANK YOU!!!
